# Developing a new multi-featured chitosan-quinoline Schiff base with potent antibacterial, antioxidant, and antidiabetic activities: design and molecular modeling simulation

**DOI:** 10.1038/s41598-023-50130-3

**Published:** 2023-12-21

**Authors:** Yasser M. Abdel-Baky, Ahmed M. Omer, Esmail M. El-Fakharany, Yousry A. Ammar, Moustafa S. Abusaif, Ahmed Ragab

**Affiliations:** 1 Chemistry Department, Faculty of Science, Al-Azhar University, Nasr City, 11884 Cairo Egypt; 2 Polymeric Materials Research Department, Advanced Technology and New Materials Research Institute (ATNMRI), City of Scientific Research and Technological Applications (SRTA-City), P. O. Box: 21934, New Borg El-Arab City, Alexandria Egypt; 3 Protein Research Department, Genetic Engineering and Biotechnology Research Institute, City of Scientific Research and Technological Applications (SRTA-City), P. O. Box: 21934, New Borg El-Arab City, Alexandria Egypt

**Keywords:** Carbohydrates, Biomedical materials, Polymer chemistry

## Abstract

A new chitosan Schiff base was developed via the reaction of chitosan (CH) with 2-chloro-3-formyl-7-ethoxy quinoline (Q) derivative. The alteration in the chemical structure and morphology of CHQ derivative was confirmed by ^1^H NMR, FT-IR spectroscopy and SEM analysis. The antibacterial activity was considerably promoted with increasing quinoline concentration up to 1 M with maximal inhibition reached 96 and 77% against *Staphylococcus haemolyticus* and *Escherichia coli*, respectively. Additionally, CHQ derivative afforded higher ABTS^·+^ radical scavenging activity reached 59% compared to 13% for native chitosan, approving its acceptable antioxidant activity. Moreover, the developed CHQ derivative can stimulate the glucose uptake in HepG-2 and yeast cells, while better inhibition of α-amylase and α-glucosidase was accomplished with maximum values of 99.78 and 92.10%, respectively. Furthermore, the molecular docking simulation clarified the binding mode of CHQ derivative inside the active site of α-amylase and α-glucosidase, suggesting its potential use as diabetes mellitus drug. The DFT calculations indicated an improvement in the electronic properties of CHQ with a lower energy band gap reached 4.05eV compared to 5.94eV for CH. The cytotoxicity assay revealed the safety of CHQ towards normal HSF cells, hypothesizing its possible application as non-toxic antibacterial, antioxidant, and antidiabetic agent for biomedical applications.

## Introduction

Over the past years, biopolymers have been extensively used in various biomedical fields due to their excellent properties, such as low-cost production, availability, biodegradability, minimal toxicity, and advantageous biological properties^1,2^. Among them, chitosan (CH) is a linear amino-polysaccharide composed of glucosamine and *N*-acetyl glucosamine units linked by *β*-(1–4) glycosidic bonds^3^. Chitosan can be obtained via simple deacetylation of chitin biopolymer, which is the main constituent of exoskeletons of crustaceans and cell walls of fungi^4^. Chemically, chitosan has three types of reactive functional groups, namely; the primary NH_2_ group at C-2, the primary and secondary OH groups at C-3 and C-6 positions, respectively^5^. In addition, the existence of active amine groups along the chitosan backbone affords its basic character and facilitates its solubility in acidic media. Owing to its outstanding biological features, including biodegradability, biocompatibility, non-toxicity, hemostaticity, and mucoadhesivness, chitosan has been widely employed in drug delivery, wound healing, and tissue engineering^6,7^. Hence, chitosan demonstrated acceptable antimicrobial, antioxidant, antiviral, and anti-inflammatory activities^8,9^, in addition to its vital role in the cytotoxic profile and targeting of cancer cells^10^. Both OH and NH_2_ functional groups of chitosan are considered the key active functional groups for its antioxidant activity since they can inhibit the reactive oxygen species (ROS) from donating hydrogen or the lone pairs of electrons^11^. Further, chitosan and its derivatives have encouraged antidiabetic profits, which are mediated over mechanisms comprising suppression of α-glucosidase and α-amylase activities, amended glucose metabolism, and reduced β-cell dysfunction^12^.

Besides, chitosan can inhibit the attack of several types of microorganisms by forming a strong protective barrier; it causes an interruption for the prokaryotic cell membrane and diffuses thoroughly to the cell nucleus accordingly^13^. Likewise, chitosan has the aptitude to stimulate the growth of the fibroblast, stop the bleeding, and inspires the migration of mononuclear/ poly morpho nuclear cells^14^. Nevertheless, the antimicrobial activity of chitosan has been gradually withstood since pathogenic bacteria are becoming resistant to antimicrobial action as a result of attained resistance genes in the DNA of the microorganism^15^. Thus, numerous efforts have been made to overcome this drawback as well as to improve its wide-range activities through numerous chemical modification techniques, such as grafting, crosslinking, quaternization, and Schiff base formation^6,16^. The former efficacious technique has been continuously getting over the top consideration due to the better biological properties of the created chitosan Schiff base derivatives compared with the pristine chitosan biopolymer^17^. This technique includes the superficial reaction of carbonyl compounds, including aldehydes and ketones with NH_2_ groups of chitosan to form the corresponding Schiff base with the imine characteristic group (–RC=N–)^17,18^. It has been reported that Schiff bases resultant from the reaction of chitosan with para-substituted benzaldehydes^19^, crotanaldehyde^20^ and salicylaldehyde^21^ unveiled superior antimicrobial activities compared with native chitosan. The authors’ previous studies also evidenced decent antimicrobial, antioxidant and anti-inflammatory activities of chitosan-4-chlorobenzaldebyde, chitosan-indole-3-carboxaldehyde, chitosan-isatin, and *N*-cinnamyl substituted O-amine functionalized chitosan^22–24^. Correspondingly, the potentials of chitosan Schiff bases have been recognized with special reference to their competencies for enhancing the inhibition rate of cancerous cell growth^25^, in addition to their satisfactory antidiabetic effects in the existence of aromatic substituent functional groups^26^.

Quinolines, are nitrogen-containing heterocyclic aromatic compounds^27^, with distinct moieties present in various biologically active natural and synthetic compounds. Substitution of the group in an appropriate site of bioactive molecules exerts a reflective pharmacological outcome. Potential applications of quinoline and its analogues are continuously spreading from anticancer drugs to practically every field of medicinal chemistry^11^. Along with their rising significance in many other fields, they have also made major contributions to society through their usage as medicinal agents, applications in veterinary and human medicine, and molecular engineering^28^. Also, their exploration contributes to discovering and developing new drugs for various diseases, addressing unmet medical needs, and improving patient outcomes^29^. Among these activities, antimicrobial, antioxidant and anti-inflammatory anticancer, antifungal, anti-plasmodium, anti-HIV, antimalarial, and anti-leishmanial activities^30–32^.

The molecular modeling is a computer experiment that controls the system's details and basic time evolution^33^. It can save time and cost in several scientific fields compared with experimental methods^34^. Over the past ten years, there has been a significant increase in the computational capabilities of molecular modeling tools, which has accelerated drug discovery^35^. It can predict the biological activity, describes the molecule's electronic properties and confirm the experimental results and are helping to predict active compounds more efficiently^36–38^. Therefore, numerous computational modeling techniques have been employed to inferior the cost and time necessary for the discovery of effective drugs. Among the most important approaches in modern drug discovery is computer-aided drug design (CAAD), which consists of a broad range of theoretical and computational approaches for predicting pharmaceutical and medicinal chemistry problems^39–41^. Moreover, molecular modeling, particularly docking simulation, provides information about the behavior and atomic-level interactions of molecules. It entails using computational techniques and algorithms to examine the target protein's three-dimensional structure. As a result, scientists may locate putative binding sites and refine drug candidates to improve their efficacy, specificity, and affinity^42^.

Realizing the medicinal importance of chitosan and quinoline, we aimed in this investigation to develop a new multi-featured chitosan Schiff base derivative based on the combination of chitosan with 2-chloro-3-formyl-7-ethoxy quinoline derivative. Till now, there is no studies reported the synthesis, bio-evaluation and molecular modelling simulation studies of this newly Schiff base derivative. The chemical structures, thermal properties, and morphological changes were inspected by several characterization tools. In comparison to pristine chitosan, the suggested chitosan-quinoline (CHQ) Schiff base is anticipated to exhibit superior performance in terms of certain biocharacteristics, such as antibacterial, antioxidant, and antidiabetic activities. The antibacterial activity of the CHQ derivative was evaluated against Gram-negative and Gram-positive bacteria, while its antioxidant activity was estimated using the ABTS decolorization assay. In addition, *α*-amylase and *α*-glucosidase inhibition studies were performed to evaluate the antidiabetic characteristic of CHQ, while the binding mode involving different types of interaction was clarified by molecular docking simulation. Moreover, a cytotoxicity assessment was also executed to ensure the safety of the synthesized CHQ Schiff base derivative. Finally, the DFT calculation including HONO, LUMO, and MEP maps were calculated to illustrate charge density distributions potentially related to biological activity.

## Materials and methods

### Materials

Chitosan powder (Mw. 100–300 KD, DD = 95%) was obtained from Qingdao Yunzhou Biochemistry Co. Ltd. (China). Phosphoryl chloride (POCL_3_, assay ≥ 97%), Dimethylformamide (DMF, assay 98%), potassium persulfate (assay 99%), 2,2 -azinobis-[3-ethylbenzothiazoline-6- sulfonic acid] diammonium salt (ABTS, purity ≥ 99), phenolphthalein (assay 98%) and m- ethoxy aniline (assay 98%)) were purchased from Sigma–Aldrich Co., (Germany). Glacial acetic acid (assay 98%), sodium acetate (assay ≥ 96%), sodium hydroxide (≥ 97%), sulfuric acid (assay 98%), ethanol (purity 99%) and hydrochloric acid (purity 37%), Sodium hydroxide (assay 98%) and Ethyl alcohol (98%) were acquired from El-Nasr Company (Egypt).

### Microorganisms

Gram positive Staphylococcus haemolyticus (*S. haemolyticus*) and gram-negative Escherichia coli (*E. coli*) bacteria were used for antibacterial evaluation studies. bacterial strains were firstly refreshed through their inoculation in Luria Betani (LB) culture medium (peptone (1%) yeast extract (0.5%) and NaCl (1%) at 37 °C for 24 h under constant shaking rate (150 rpm).

### Synthesis of chitosan–quinoline Schiff base (CHQ)

**Figure 1:**
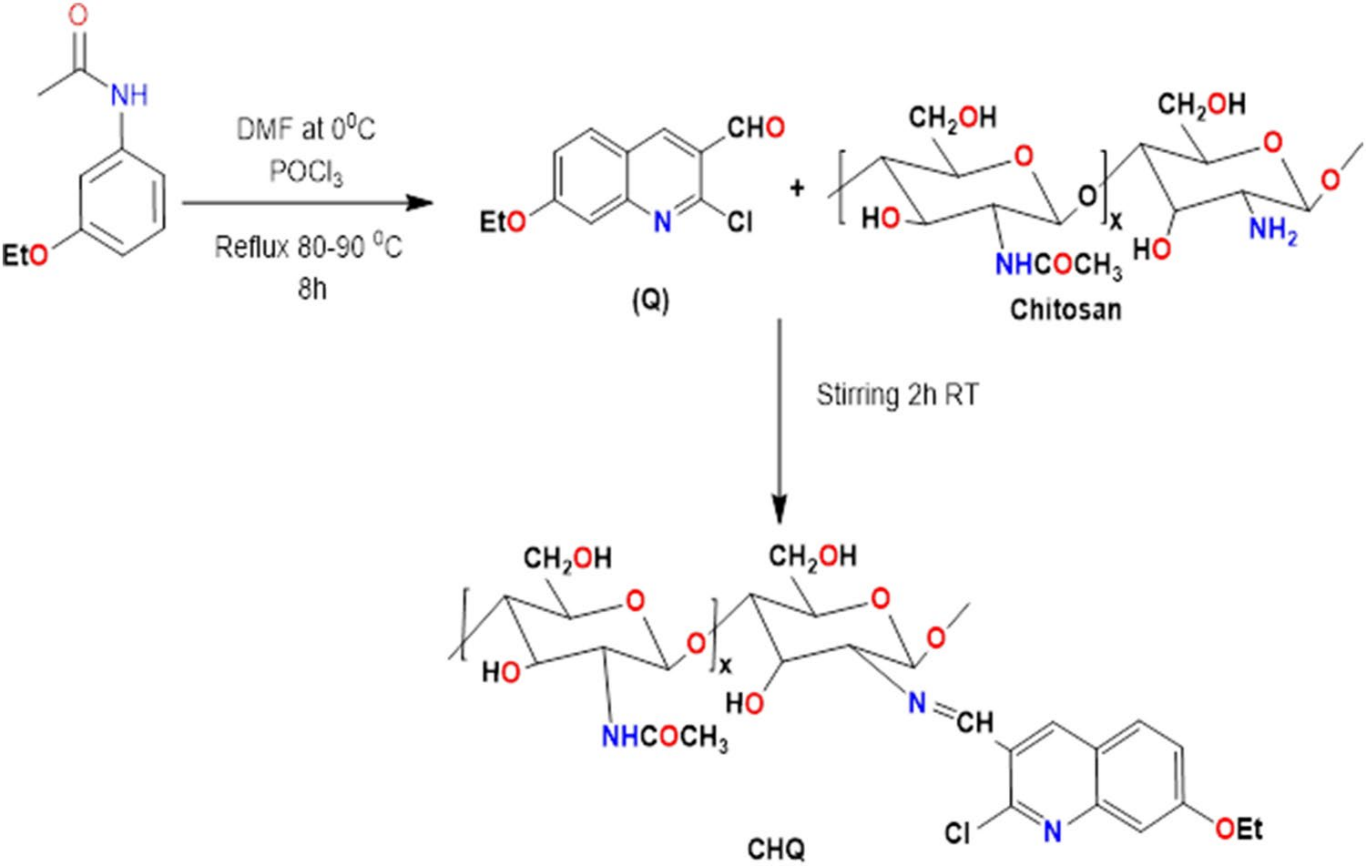
Synthetic pathway for synthesis of chitosan-quinoline Schiff base derivative.

Before synthesizing the Schiff base derivative, 2-chloro-3-formyl-7-ethoxy quinoline was synthesized according to the previously reported method using the Vilsmeier reaction^43^. A known weight of chitosan (1 g) was dissolved in acetic acid (2%; w/v) solution under stirring at room temperature to obtain a homogenous solution with a final concentration of 2%; w/v. After that, the quinoline derivative was dissolved in 15 mL of ethanol and subsequently added to the chitosan solution under continuous stirring conditions. The reaction mixture was stirred for 2 h at room temperature, while a light-yellow colour solution was observed, which refers to the formation of chitosan–quinoline Schiff base derivative (CHQ). An excess of sodium hydroxide solution (5%) was added to the mixture precipitate CHQ derivative, which was followed by filtration and washing several times using distilled water and ethanol to eliminate the excess of alkali and unreacted quinoline derivative. Finally, the purified CHQ Schiff base derivative was dried overnight in a vacuum oven at 65 °C. Three different ratios of CHQ derivative were prepared with final CH: Q of 1.0: 0.25, 1.0: 0.5, and 1 M, which were coded as CHQ (0.25), CHQ (0.5), and CHQ (1), respectively, in addition to pure chitosan (CH). The synthetic route for synthesizing CHQ derivative was illustrated in Fig. 1.

### Instrumental characterization

The chemical structure of CHQ derivative was examined at 400 MHz using nuclear magnetic resonance (1H NMR, Bruker DRX-400 spectrometer, USA) and Fourier transform infrared spectroscopy (FTIR, Model 8400 S, Shimadzu, Kyoto, Japan). The FTIR spectra were recorded at 4000–500 cm^−1^ using 5 mg of dried sample and KBr discs. The morphological properties were investigated a Scanning electron microscope (SEM; Model JSM 6360 LA, Joel, Japan). Prior to SEM analysis, the tested dry sample was spread out on a double-sided conducting adhesive tape, fixed on a metallic stub, and then coated with a thin layer of gold.

### Determination of degree of substitution and ion exchange capacity

Degree of substitution (DS) of the developed CHQ Schiff base derivative was estimated based on the 1H NMR spectrum following the Eq. (1) ^44^. On the other hand, ion exchange capacity (IEC) measurements were achieved via immersing a definite quantity of tested samples into 0.1 M of H_2_SO_4_ solution and kept for 2 h. Next, the mixture was filtered, and a filtrate was titrated against a standard solution of NaOH (0.1 N). Similarly, control titration without the addition of the tested sample was also performed, while ion exchange capacity (IEC) was calculated according to the following Eq. (2) ^16^:

1 $${\text{DS }}\left( {\text{\% }} \right) = \frac{{{ }\left( {{\text{H}}10} \right)}}{{\left( {{\text{H}}2} \right)}} \times 100$$

where H10 and H2 represent the peaks area of the immine proton and the proton H2, respectively.

2 $${\text{IEC }}\left( {{\text{meq}}.{\text{g}}} \right) = \frac{{{ }\left( {{\text{V}}2 - {\text{V}}1} \right){\text{N}}}}{{\text{w}}} \times 100$$

where V_2_ and V_1_ are the volumes of NaOH required for complete neutralization of H_2_SO_4_ in the absence and in the presence of the examined sample, respectively. The N is the normality of NaOH solution and w is the weight of examined sample taken for analysis.

### Bio-evaluation studies

#### Antibacterial activity assay

Two approaches were performed for screening and evaluating the antibacterial activity of neat CH and CHQ Schiff base derivatives: broth dilution and agar-well diffusion bioassays^45^. For broth dilution assay, the previously refreshed bacterial suspensions were diluted up to 100 times using LB broth medium (1%). Next, 0.1 mL of diluted bacteria suspension was cultured in 10 mL of liquid peptone medium and followed with the addition of the tested sample. The inoculated medium remained shacked for 24 h at 37 °C, while the inhibition of bacterial growth was determined using visible-spectroscopy at 620 nm depending on Eq. (3). Besides, the minimal inhibitory concentration (MIC) was accomplished by the addition of pure spore suspension of each tested bacteria (2 mL of 10^6^ cells/mL) into sterile test tubes containing 50 µL of different concentrations of examined CHQ Schiff base derivative (25–200 µg/mL). The tubes were then incubated at 37 °C for 24 h. At the same time, the MIC results were represented graphically as a function of the optical density (Eq. 4) of bacterial growth, which was measured at 620 nm using visible-spectroscopy at 620 nm following equations:

3 $${\text{Inhibition }}\left( {\text{\% }} \right) = \frac{{{\text{A}}_{{\text{a}}} - {\text{A}}_{{\text{b}}} }}{{{\text{A}}_{{\text{a}}} }} \times 100$$

where A_a_ and A_b_ are the absorbance in the absence and in the presence of examined sample, respectively.

4 $$\mathrm{Inhibition }(\mathrm{\%})=\frac{\mathrm{ODn }-\mathrm{ ODs}}{{\text{ODn}}}\times 100$$

where ODs and ODn are the optical densities of the normal and inhibited bacterial growth, respectively.

On the other hand, the second approach (i.e., agar-well diffusion method) was conducted via spreading of 100 μL of *S. haemolyticus* and *E. coli* suspensions onto the LB-agar plates using a sterile glass spreader, while the colony forming unit of cultures was 2 × 10^8^ CFU/mL. The plates were allowed to dry for 3–5 min, since a sterile cork borer was used for forming wells of 5 mm diameter on the surface of agar plates. Subsequently, 50 µL of the tested sample (10 mg/mL) were soaked into each well, and the plates were incubated at 37 °C for 24 h. After incubation, the diameter (mm) of the inhibition zone was estimated, while the experiments were performed in triplicate. Besides, the microtiter plate method for determining the MIC for various antimicrobial agents.

#### Antioxidant activity assay

Antioxidant activity assessment of the synthesized CHQ derivative was performed according to the reported ABTS assay^46^. The ABTS radical cation (ABTS· +) was generated by the reaction of potassium persulfate (3.3 mg/5 mL) in H_2_O (5 mL) with ABTS (17.2 mg). The resultant bluish-green radical cation solution was kept in the dark below 0 °C overnight. Afterward, an accurate 1 mL of the previous solution was diluted with 0.2% of acetic acid solution to reach a final volume of 60 mL. A definite volume (0.1 mL) of the examined sample (1%, w/v) was added into a glass test tube containing 2 mL of diluted ABTS· + solution, followed by dark incubation at room temperature for 30 min. The radical scavenging (%) was assayed as a function of measuring the absorbance at 734 nm using a spectrophotometer (UV-1200S, China), as represented by the following Eq. (5):

5 $${\text{Radical}}\;{\text{scavenging}}\left( \% \right) = \left[ {\left( {{\text{A}}_{{{\text{control}}}} {-}{\text{A}}_{{{\text{sample}}}} } \right)/{\text{A}}_{{{\text{control}}}} } \right] \times {1}00$$

where A _control_ and A _sample_ represent the absorbance of ABTS solution with and without the tested sample, respectively.

#### In vitro diabetic inhibition studies

##### Glucose utilization in HepG-2 and yeast cells

The assessment of glucose utilization in HepG-2 cells was performed according to the reported method^47^. HepG-2 cells (10^4^ cells/ well) were cultured in a sterile culture plate (96-well) and incubated in a humidified incubator (5% CO_2_) at 37 °C overnight. Different concentrations of examined CH and CHQ derivatives (5–50 µg/mL) were added and subsequently followed by incubation for two days at the same conditions. The culture medium was relieved with RPMI-1640 medium comprising 8 mM of glucose and 0.1% of BSA solutions and incubated again for 3 h at 37 °C. To assess glucose concentration in the medium, 10 μL of the medium was transferred into a 96-well plate containing glucose oxidase reagent followed by incubation for 15 min at 37 °C. The optical densities were measured at 492 nm using an ELISA micro-plate reader. The glucose uptake (%) by HepG-2 cells was calculated based on Eq. (7). On the other hand, to estimate the glucose uptake by yeast cells, the suspension of yeast cells was firstly washed with distilled water many times using centrifugation at 4000 rpm. The examined samples (5- 50 µg/mL) were added to 180 µL of glucose solution (25 mM) and incubated for 10 min at 37 °C. Thereafter, the reaction was initiated by adding yeast suspension (20 µL), followed by a well-shaking and incubation at 37 °C for 3 h. Later, the mixtures were centrifuged (3000 rpm for 5 min), while the glucose uptake (%) by yeast was determined in the supernatant according to Eq. 6. Likewise, untreated HepG-2/or yeast cells were served as a negative reference, while cells treated with berberine-drug at the same concentrations were served as a positive reference.

6 $${\text{Gglucose uptake }}\left( {\text{\% }} \right) = \left( {{\text{OD}}_{{\text{S}}} - {\text{OD}}_{{\text{C}}} } \right) \times 100$$

where OD_C_ and OD_S_ ore the optical densities in absence (control) and in presence of tested sample, respectively.

##### α-Amylase and α-glucosidase inhibition assays

*Α***-**amylase inhibition by CH and CHQ derivatives were investigated according to the author’s previous work^48^. Briefly, accurate concentration (0.25–8.0 mg/mL) of examined samples was added to 1 mL of α **-**amylase enzyme solution (20 mM/ phosphate buffer pH 7.2). The mixture was incubated for 10 min at 37 °C, followed by addition of 1 mL of water-soluble starch solution (0.5%) and incubated for another 10 min. The reaction was stopped by adding 0.5 mL of 1 M HCl, followed by adding iodine solution. Phosphate buffer (pH 7.2) was employed as blank and acarbose was served as a positive control. The optical density (OD) was assessed at 660 nm using a visible-spectroscopy and the inhibitory percentage of α-amylase activity was calculated using the Eq. (7). On the other hand, α-Glucosidase inhibition assay was performed by adding 50 μL of tested sample base derivative (0.25–8.0 mg/mL) was added to 1 mL of α-glucosidase solution (50 μg/mL/phosphate buffer pH 7.2). After incubation for 5 min at room temperature, 100 μL of ρ-nitrophenyl-α-D-glucoside (10 mM) solution was added and followed by incubation at 37 °C for another 20 min. 250 μL of 100 mM sodium carbonate was subsequently added to terminate the reaction, while the OD was determined at 405 nm. Phosphate buffer was also used as a blank, while Curcumin drug was employed as a positive control. The α-glucosidase inhibition (%) was assayed also using the Eq. (7). The IC_50_% values (the concentration of the compounds that caused inhibition of 50% of α-amylase and α-glucosidase activities) were calculated by using graph pad prism 6.0 software.

7 $${\upalpha } - {\text{amylase }}\left( {{\text{or}}\;{\upalpha } - {\text{Glucosidase}}} \right)\;{\text{inhibition}}\left( \% \right) = \left[ {1 - \frac{{OD_{S} - OD_{B} }}{{OD_{C} }}} \right] \times 100$$

where OD_S_, OD_B_ and OD_C_ represent the optical densities in presence of examined sample, blank and control, respectively.

### Cytotoxicity assay

To evaluate the cytotoxic impact of CH and CHQ Schiff base derivative, the MTT method was applied using a normal HSF (normal somatic skin cells) cell line^49^. The HSF cells were cultured in two sterile 96-well tissue plates, followed by incubation overnight. The cells were then treated with CH and CHQ Schiff base derivative (25–200 mg). After incubation in CO_2_ incubator (5% CO_2,_ 85% humidity) at 37 °C for 48 h, the treated and untreated cells were washed with fresh medium (200 μ), followed by adding MTT (0.5 mg/mL dissolved in culture medium) and incubated for further 3 h. The MTT medium was substituted with DMSO (200 μL/well) to solubilize the crystal (purple formazan), while the optical density (OD) was assayed at 590 nm using an ELISA microplate reader. The cell viability (%) was calculated using the following Eq. (8):

8 $${\text{Cell viability}}\left( {\text{\% }} \right) = \frac{{{\text{OD}}_{{{\text{treated}}}} }}{{{\text{OD}}_{{{\text{reference}}}} }} \times 10$$

### Molecular docking simulation

The binding mode of the newly designed CHQ Schiff base derivative in addition to native chitosan (CH), and quinoline derivative (Q) were performed inside the active site of α-amylase (PDB: 2QV4) and α-glycosidase (PDB: 3W37) using Molecular Operating Environment (MOE)^38,50^. The docking score energy is represented by binding energy (S) with the negative values. The two proteins α-amylase (PDB: 2QV4) and α-glycosidase (PDB: 3W37) were obtained from the protein data bank. The water molecules were removed, and the proteins with a co-crystallized ligand were exposed to minimize the energy using forcefield MMFF94X with a gradient of 0.05 kcal/mol. The structure of the tested samples was built using chembiodraw 2014, and then all hydrogen atoms were added, while the structures were minimized at the same force field and finally saved. The active site of α-amylase (PDB: 2QV4) and α-glycosidase (PDB: 3W37) were generated according to the previously reported methods^51,52^. For α-amylase (PDB: 2QV4), the co-crystallized ligand (acarbose) (Fig. S1 (A, B)) exhibited binding energy S = − 9.62 kcal/mol with RMSD = 1.095 Å through two hydrogen bonds side-chain acceptors (His299 and His201) and five hydrogen bonds side-chain donor (Asn105, Asp300, and Glu233. For α-glycosidase (PDB: 3W37), the co-crystallized ligand (acarbose) (Fig. S2 (A, B)) displayed binding energy S = − 8.69 kcal/mol with RMSD = 2.05 Å through eight hydrogen bonds side-chain donor and two hydrogen bonds side-chain acceptor.

### DFT calculation

The DFT calculation was carried out by using the Becke-three-parameters-Lee–Yang–Parr hybrid functional (B3LYP) and standard basis set 6-31G(d) using geometrical optimization in Gaussian 09 and GaussView 6.0 according to previously reported method^53^. The frontier molecular orbitals (FMO), molecular electrostatic potential (MEPs), and chemical reactivity descriptors values as ionization potential (i); electron affinity (EA); electronegativity (X); chemical hardness (ɳ); chemical softness (S); chemical potential (µp); electrophilic index (ω) that calculated according to previously reported method^50,54^.

### Statistical analysis

All experiments were performed in triplicate (n = 3), and the results were expressed as the mean standard deviation (± SD).

### Ethical approval

This research was approved by the City of Scientific Research and Technological Applications (SRTA-City, Egypt). Moreover, the entire analysis was performed in accordance with the relevant guidelines.

## Results and discussion

### ^1^H NMR and IEC analysis

**Figure 2:**
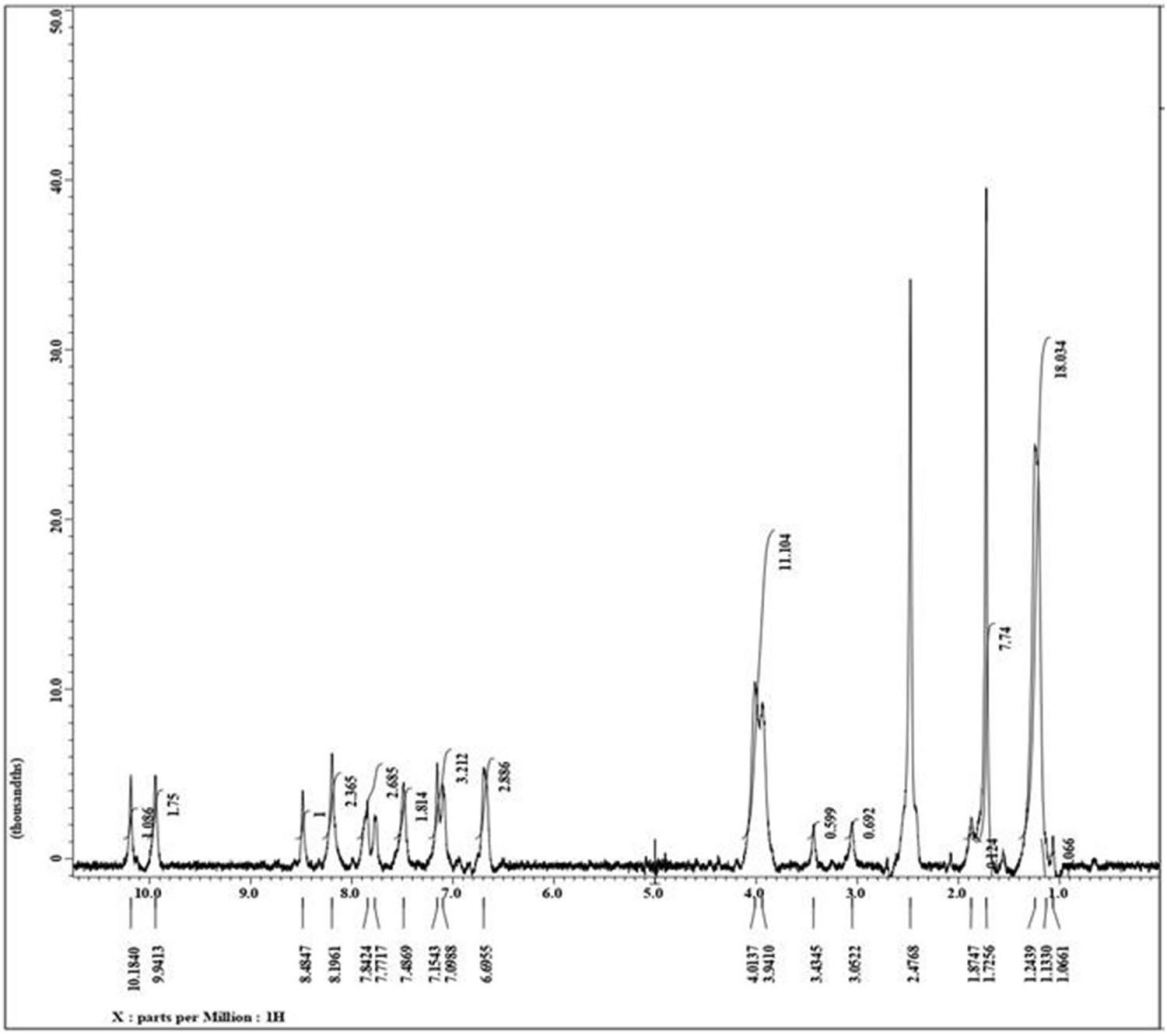
H^1^ NMR of CHQ Schiff base derivative.

The ^1^H NMR spectrum of the CHQ Schiff base (Fig. 2) displayed significant signals at *δ* 1.34 and 4.01 ppm related to the ethoxy group of 7-ethoxy quinoline derivative. Additionally, three singlet signals appeared at *δ* 10.18, 9.94, and 8.48 ppm attributed to the NH of *N*-acetylglucosamine (GlcNAc), methine group (CH=N), and quinoline-H4^55^. In addition, the aromatic protons of quinoline moiety displayed as two doublets and one singlet signal at *δ* 7.48, 7.84, and 8.19 ppm. The upper field revealed several overlapping signals from *δ* 1.72 to 3.05 ppm, referring to protons of glucopyranose ring and methyl of *N*-acetylglucosamine (GlcNAc), while anomeric proton (H1) appears at δ 6.69 ppm. These results designate the evidence of the formation of CHQ derivative. Furthermore, the degree of the substitution (DS) was determined based on the ratio between the integrated area of the proton signal of the imine group and signal of the proton (H-2) of the GlucN unit^44,56^. The results obtained from the 1H NMR spectrum signified that the DS increased with increasing the quinolone ratio in the synthetized Schiff base derivative. Hence, the calculated DS (%) values were recorded as 32.0, 63.5 and 78.83% for CHQ (0.25), CHQ (0. 5) and CHQ (1) Schiff base derivatives, respectively.

Besides, ion exchange capacity (IEC) can be taken as an indicator for the evidence of the Schiff base formation occurrence. The results referred that the IEC value was significantly decreased with increasing quinoline concentration involved in the reaction. Therefore, pure chitosan recorded the highest IEC value of 12.86 ± 0.43 m_eq_/g, which was decreased with increasing quinolone ratio in the formed Schiff base. Therefore, CHQ (0.25), CHQ (0.5) and CHQ(1) recorded 11 ± 0.85, 8.77 ± 0.63 and 4.46 ± 0.37m_eq_/g, respectively. These observations could be ascribed to the consumption of the primary NH_2_ groups distributed on the chitosan polymer backbone through coupling with formyl-quinoline moiety for Schiff base formation^57^. The gained IEC results agreed with the obtained behavior of the substitution degree.

### FTIR spectra

**Figure 3:**
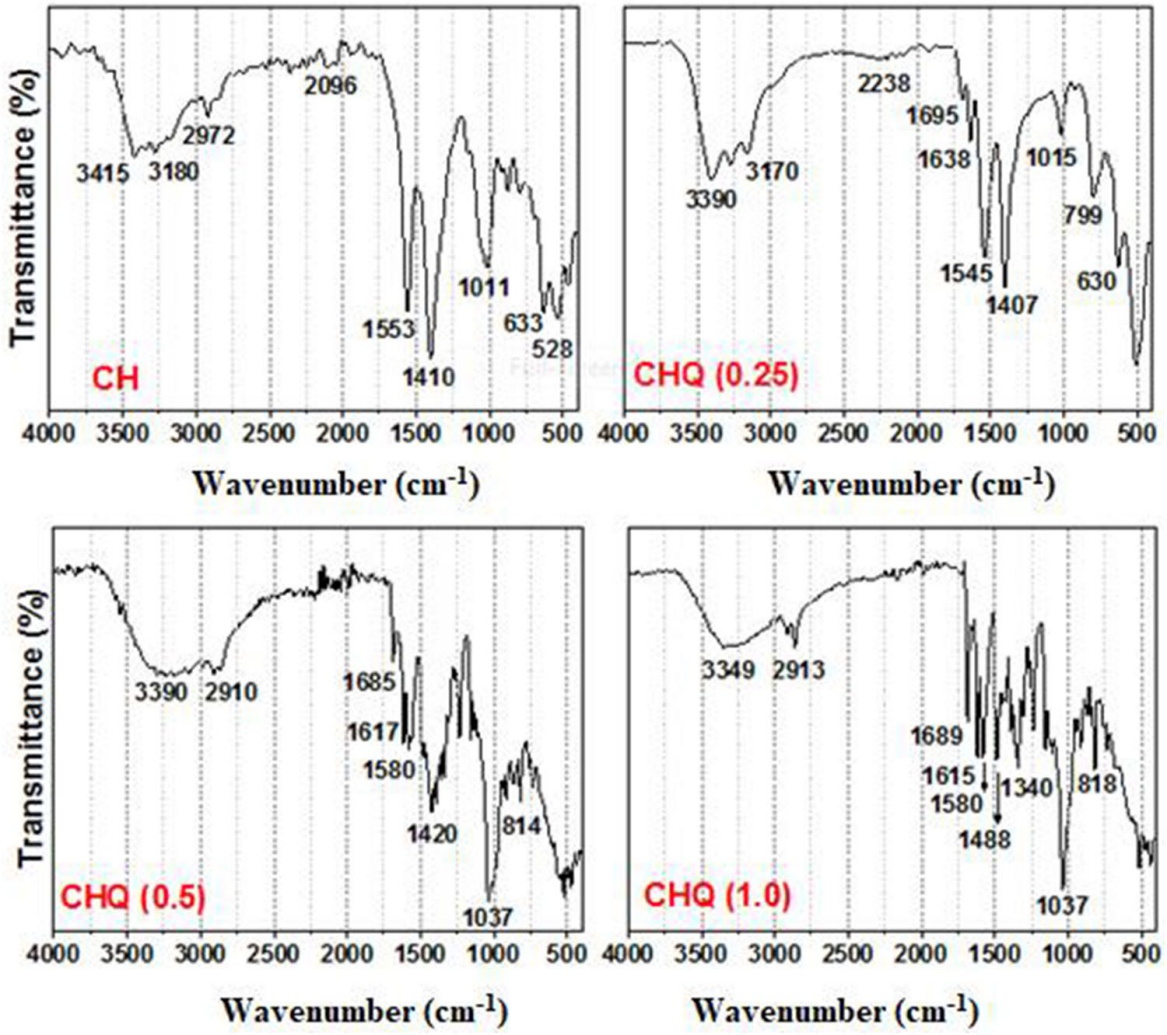
FTIR spectra of chitosan (CH) and CHQ Schiff base derivative with different quinoline concentrations.

The IR spectra depicted in Fig. 3 were performed to get more details regarding the chemical structures of chitosan and the newly synthesized CHQ derivative. In the IR spectrum of chitosan (CH), the broad band at 3500–3000 cm^−1^ corresponds to the stretching vibration of OH and NH_2_ modes and those involved in the hydrogen bonds, while a sharp band at 1410 cm^−1^ indicated the presence of hydroxyl groups (O–H bending)^58^. The weak absorption peak at 2972 cm^–1^ is related to C–H stretching. The absorption band at 1011 cm^−1^ corresponds to C–C, C–O, and C–O–C glycosidic bonds^59^. Furthermore, the formation of derivative (CHQ) has confirmed the presence of characteristic bands at 3390–3190, 2998–2910, 1695–1685, 1615–1638, 1580–1545, 1488–1407, and 1037–1015 cm^−1^ corresponding to OH + NH, C–H stretching (CH_2_), carbonyl group (C=O), imine group (C=N), C=C of the aromatic ring, OH bending, and carbonyl group stretching (C=O), respectively^60^. Additionally, it was observed that the intensity of the carbonyl and azomethane groups increased with an increase in the concentration of quinoline moiety, confirming the successful formation of CHQ Schiff base derivative.

### SEM analysis

**Figure 4:**
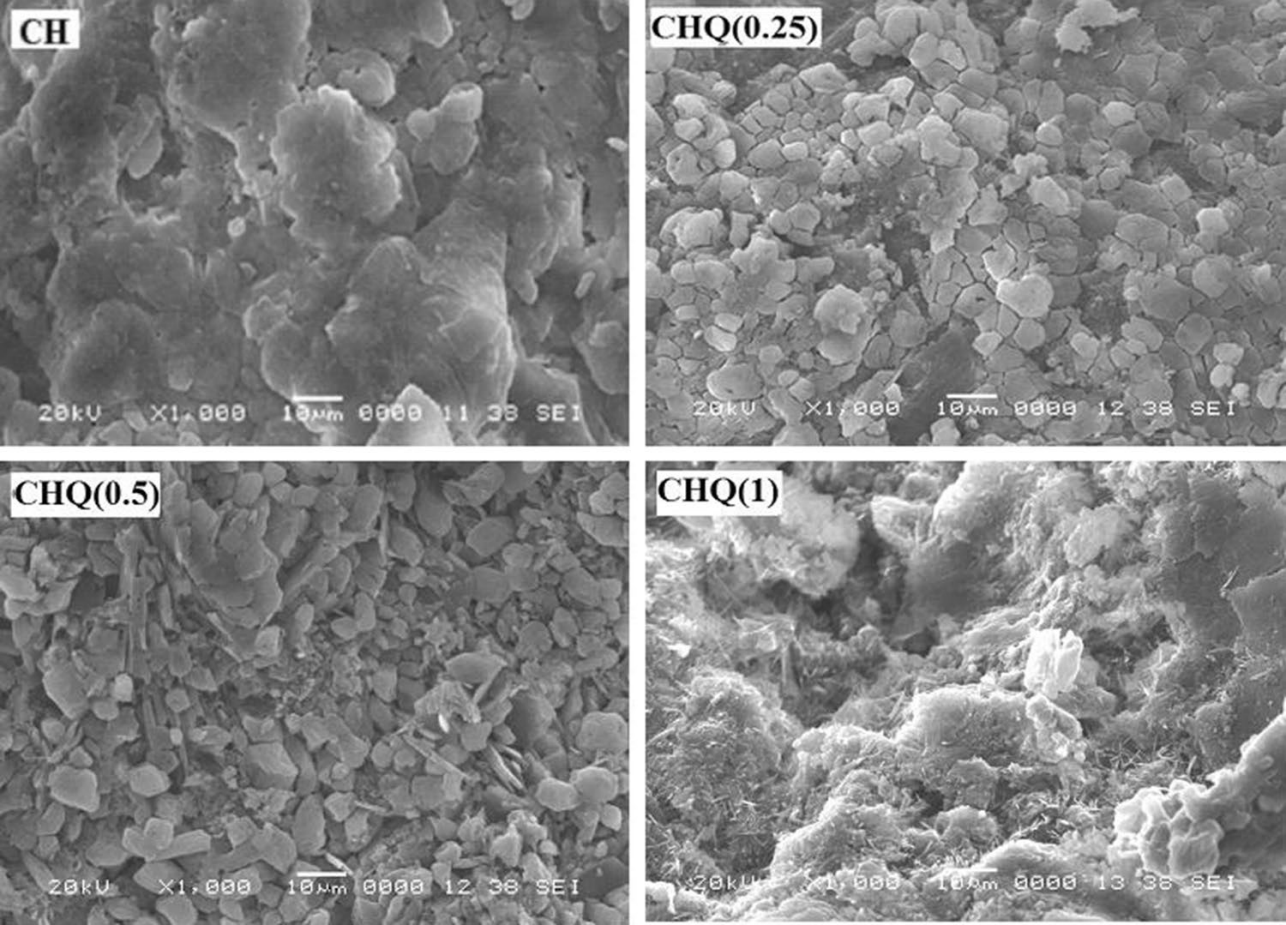
SEM images of CHQ Schiff base derivative with different quinoline concentrations.

Using a scanning electron microscope (SEM), the surface morphological analysis of pure CH and CHQ derivatives were studied, as shown in Fig. 4. The SEM analysis showed a dramatic increase in surface roughness with an increase of quinoline scaffold concentration in the prepared CHQ Schiff base, where get maximum roughness in case of CHQ (1). These findings might be explained by the changes in the internal structure of the native chitosan resultant from the difference in polarity between CH and quinoline derivative^22^. Introducing the quinoline moiety into the chitosan backbone gives some hydrophobic nature to the chitosan Schiff base. This modification induced a change in the internal forces between polymer backbone chains (i.e., hydrogen bond, Van der Waals forces, etc.). Besides, it may cause changes in the solid-state crystallinity of the modified polymer^61^.

### In vitro bio-evaluation

#### Evaluation of antibacterial activity

Bacterial infection causes a lot of complications, including delays in the wound healing process, and in sometimes leads to death. Mutations of microorganisms to resist the activity of antibacterial agents have prompted researchers to develop new efficient materials with higher potential activity against the new version of pathogenic microorganisms^62,63^.

**Figure 5:**
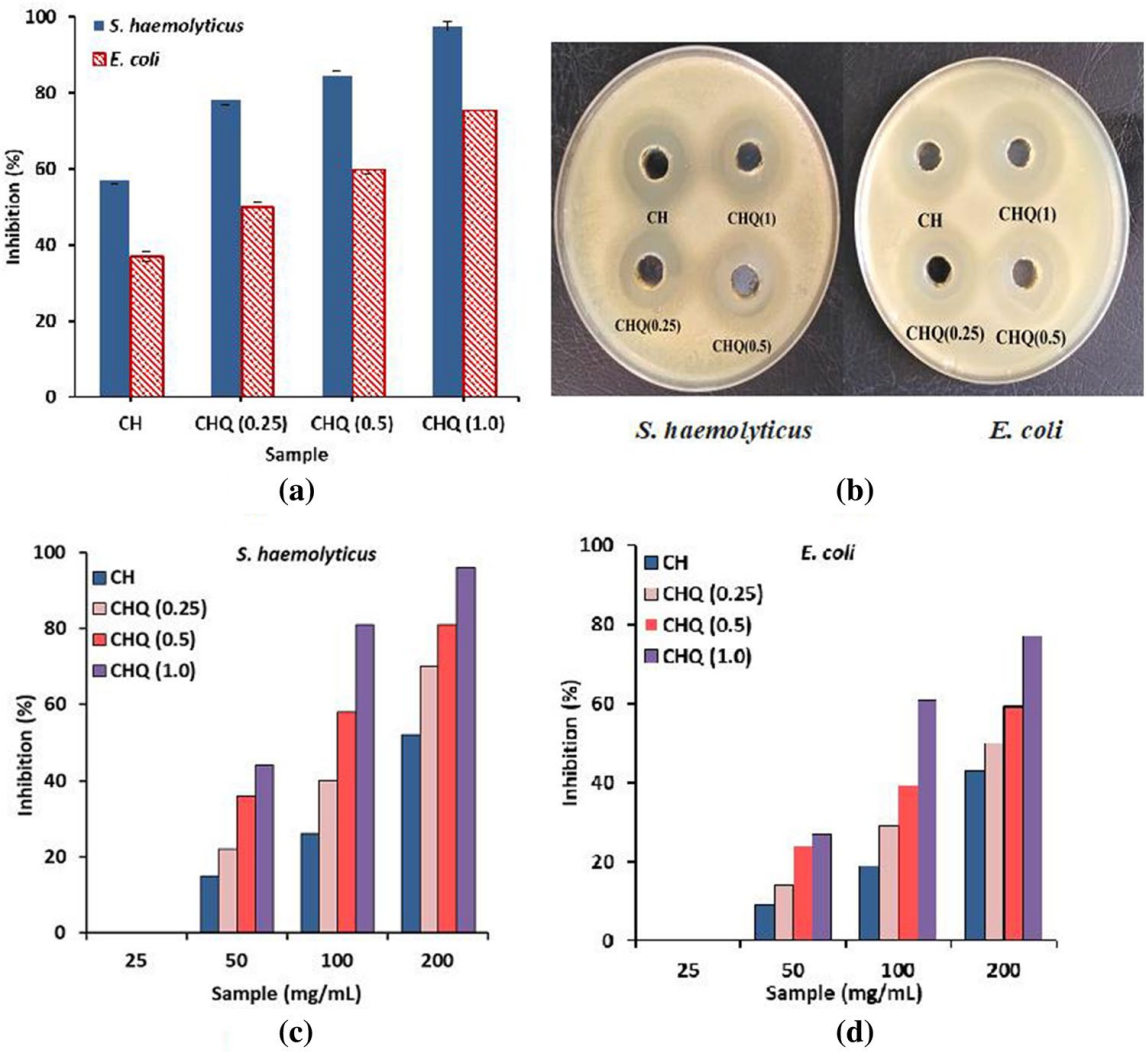
Inhibitory effect of chitosan (CH) and chitosan-quinoline schiff base (CHQ) derivative with different concentrations of quinoline against *S. haemolyticus* and *E. coli* using (**a**) Broth dilution method and (**b**) Agar-well diffusion method. (**c**) MIC results against *S. haemolyticus* and (**d**) MIC esults against *E. coli*.

The inhibitory effect of the newly synthesized chitosan-quinoline derivative (CHQ) was evaluated for its antibacterial activity against one gram-positive bacteria (*S. haemolyticus*) and other gram-negative bacteria (*E. coli*) using the Broth dilution method. Figure 5a clarified that the chitosan-quinoline derivative demonstrated higher antibacterial activity than chitosan itself, while this activity was increased with increasing quinoline moiety concentration in the synthesized Schiff base compound. Therefore, maximal inhibition of 97.55 and 75.44% were accomplished by CHQ (1) sample against *S. haemolyticus* and *E. coli* bacteria compared to 57.3 and 36.9% for pure chitosan (CH). The significant increase in the antibacterial activity of CHQ derivative can be attributed to the generation of new imine groups in addition to the introduction of quinoline scaffold that had a hydrophobic nature along the chitosan backbone. Moreover, the results refereed also that the CHQ derivative showed better activity against gram-positive bacteria (*S. haemolyticus*) compared to gram-negative bacteria (*E. coli*). These observations were further proved via the agar-well diffusion approach, as displayed in Fig. 5b and Table 1. The inhibitory effect was measured based on the clear zone surrounding the circular solution. As expected, a significant enlargement in the inhibition zone was noticed with increasing the quinoline content in the synthesized Schiff base. The inhibition zones of pure chitosan (CH) in the case of *S. haemolyticus* and *E. coli* bacteria were 33.5 ± 0.23 and 28 ± 0.42 mm, respectively, which were gradually increased up to 37.0 ± 0.45 and 32.5 ± 0.37 mm in the case of a sample containing the highest quinoline concentration (CHQ (1)). This activity may be attributed to forming of a new Schiff base bond between the quinoline derivative and chitosan polymer after the modification process.

**Table 1:** Estimated diameter of the inhibition zones of chitosan-quinoline derivative (CHQ) using Agar-well diffusion method.

Inhibition zone (mm)		
Sample code	*S. haemolyticus*	*E. coli*
CH	33.5 ± 0.23	28.0 ± 0.42
CHQ (0.25)	34.0 ± 0.53	30.0 ± 0.65
CHQ (0.5)	35.0 ± 0.65	31.5 ± 0.24
CHQ (1.0)	37.0 ± 0.45	32.5 ± 0.37

Although the mechanism of the antibacterial action of chitosan and its derivative is not yet certainly understood, three proposed mechanisms have been reported to explore this aspect^64,65^. The first mechanism which considered the most acceptable one for this study as it involves the electrostatic interaction between the positive charges of chitosan/or its derivative and the negative charges on the cell wall of bacteria, which incites leakage of intracellular ingredients (i.e., amino acids, proteins, glucose, and lactic dehydrogenase) because of disruption of the bacterial cell membrane. The second mechanism is associated with the chelating capability of chitosan towards metal ions such as Ca^2+^, Mg^2+^_,_ and Zn^2+^, which are essential for the growth of bacteria in addition to their role in the metabolic paths, such as spore formation in the gram-positive bacteria. The third mechanism demonstrates the diffusion of the low molecular weight chitosan into the nuclei of microorganisms, which bind with DNA, suppress the mRNA expression followed by stopping the protein synthesis, resulting in the death of the bacterial cells. Additionally, introduction of hydrophobic characteristic groups resultant from the reaction of quinoline molecule with chitosan would improve the interactions with peptidoglycan and lipoprotein in the outer membrane of bacterial cell wall. Hence, these interactions result in a block of the feeding channels responsible for exchanging electrolytes and nutrients and consequently, the inhibition activity of bacterial growth increases^57^.

#### MIC estimation

Minimum inhibitory concentration (MIC) of bioactive materials is considered one of the most vital parameters for using antibacterial agents to lessen their unfavorable properties. Here, we have examined the lowest concentration that could suppress the bacterial growth using different concentrations of chitosan and the synthesized CHQ derivative (25–200 µg/mL) as presented in Fig. 5c and d. It can be detected that all samples didn’t display any activity against *S. haemolyticus* and *E. coli* bacteria at the lowest concentration of 25 µg/mL. With increasing concentration of the examined sample up to 200 µg/mL inhibition activity was gradually increased. Furthermore, the result indicated that hybridization between chitosan and quinoline displayed the highest MIC values. The MIC values were augmented by using 200 µg/mL of the sample with increasing quinoline ratio. Maximal values of 96 and 77% were perceived by CHQ (1) derivative against *S. haemolyticus* and *E. coli* bacteria, respectively, compared to only 52 and 43%, which were recorded by pure chitosan (CH).

Besides, the higher activity against Gram-positive bacteria (*S. haemolyticus)* than Gram-negative bacteria (*E. coli)* could be attributed to the existence of thick peptidoglycan at the cell wall surface of Gram-positive bacteria including teichoic acid molecules with negative charges. These charges can bind with the positively charged groups of chitosan and its derivative^23^ through the electrostatic interactions, while materials can diffuse inside the cells to inhibit protein synthesis, resulting in the destruction of bacterial cells. In contrast, the presence of multi-barrier membranes in Gram-negative bacteria would hamper the diffusion of examined materials inside the cells, which include the outer membrane with a hydrophobic moiety, peptidoglycan layer, and cell membrane. These antibacterial manners are consistent with the other reported studies^8,66^. Overall, we could conclude from these findings that the synthesized CHQ Schiff base derivative established better antibacterial potential compared to pristine chitosan, suggesting its possible application as antibacterial agent.

#### Antioxidative activity

The antioxidant properties of chitosan and the synthesized CHQ derivative were assessed by adapting the ABTS assay. The results depicted in Table 2 signified that the CHQ derivative exhibited higher ABTS^·+^ radical scavenging activity than pure chitosan, while a dramatic increase in the antioxidant activity was observed with increasing concentration of quinoline in the CHQ Schiff base derivative^11^.

**Table 2:** Antioxidant activity of chitosan-quinoline derivative (CHQ) represented by radical scvenging (%).

Sample code	Radical scavenging (%)
CH	13 ± 0.54
CHQ (0.25)	29 ± 0.62
CHQ (0.5)	41 ± 0.46
CHQ (1)	56 ± 0.72

The color of ABTS^·+^ was reduced by 59% using CHQ (1) compared to only 13%, which was recorded by native chitosan. The increase in the antioxidant action could be explained by the presence of more electron donating atoms, such as nitrogen and oxygen, along the hybrid structure of the CHQ derivative, which enhance its aptitude to scavenge ABTS^·+^ radicals^59^.

#### Antidiabetic evaluation

**Figure 6:**
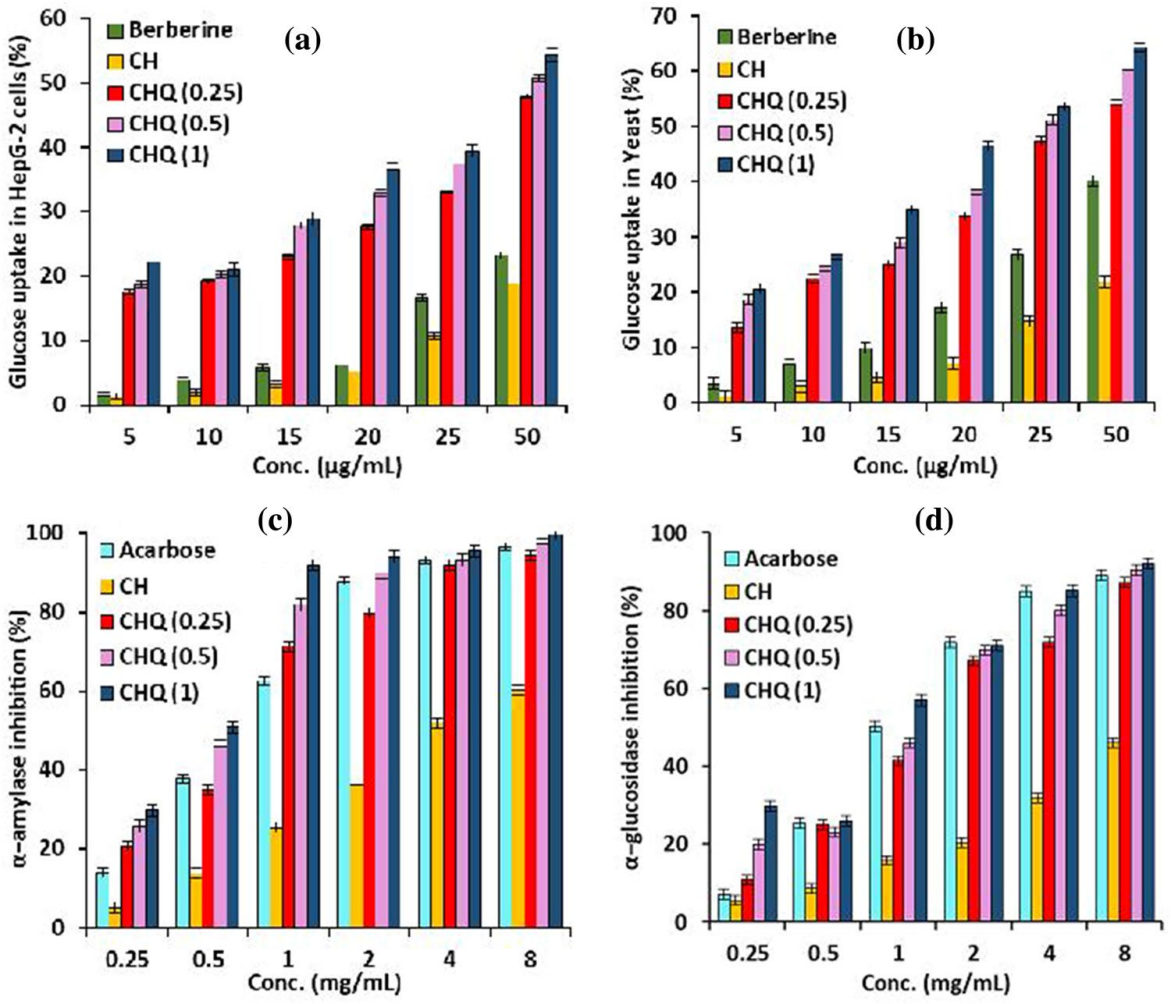
Effect of chitosan and CHQ derivative on (**a**) glucose uptake in HepG-2 cells, (**b**) glucose uptake in Yeast cells, (**c**) inhibition of α-amylase and (**d**) inhibition of α-glucosidase. Data were expressed as mean ± SEM (n = 3).

Figure 6a and b displayed the impact of different CHQ derivative concentrations on glucose utilization in HepG-2 cells and Yeast cells. The results clarified that the CHQ derivative had the aptitude to cause a noticeable increase in glucose uptake percent in both HepG-2 cells and yeast cells at all tested concentrations (5–50 µg/mL) in a concentration-dependent manner compared to pure chitosan (CH). Notably, the glucose uptake values were augmented with increasing quinoline concentration in the synthesized CHQ derivative. Therefore, maximal values of 54.29% (in HepG-2 cells) and 64.19% (in yeast cells) were recorded by CHQ (1) sample at the highest concentration of 50 µg/mL compared to 18.8, 21.8%, and 23.18, 40.18% which were recorded by pure chitosan (CH) and Berberine-drug. The gained results suggested that the CHQ derivative could effectually stimulate the glucose uptake in both HepG-2 cells and yeast cells, in addition to controlling the blood glucose levels via enhancing the effective glucose uptake.

On the other hand, the results depicted in Fig. 6c and d revealed that the CHQ derivative exerted the greatest inhibitory action at the concentration range 0.25–8.0 mg/mL. At the highest concentration of 8.0 mg/mL, the most potent α-amylase and α-glucosidase inhibitory activities were recorded by a sample containing the highest quinoline concentration (CHQ (1)) with maximal values reached 99.78 and 92.10% compared to 60.22 and 46.10% for pristine CH. Besides, Table 3 showed that the concentrations of the examined CHQ derivative that can inhibit about 50% of α-amylase activities (IC_50%_) were detected in the range 0.513–0.694 mg/mL compared to 2.325 and 0.821 mg/mL for native CH and acarbose-drug, respectively. In addition, about 1.008–1.267 mg/mL was needed from CHQ derivative for the inhibition of 50% of α-glucosidase compared to 9.326 and 1.070 mg/mL for native CH and acarbose-drug, respectively. These results confirmed that introducing quinoline moiety to chitosan via Schiff base formation had a positive impact on the inhibition of both α-amylase and α-glucosidase^12,48^.

**Table 3:** IC_50%_ values for the inhibition of α-amylase and α-glucosidase by CH and CHQ derivative with different quinoline concentrations.

IC_50%_ (mg/mL) *		
Sample	α-amylase	α-glucosidase
Acarbose	0.821 ± 0.026	1.070 ± 0.062
CH	2.325 ± 0.121	9.326 ± 0.456
CHQ (0.25)	0. 694 ± 0.087	1.267 ± 0.026
CHQ (0.5)	0. 560 ± 0.038	1.121 ± 0.041
CHQ (1)	0. 513 ± 0.039	1.008 ± 0.033

*All data are presented as mean ± SEM of triplicates.

The anti-α-amylase activity of the chitosan and its synthesized derivatives were rarely studied. Previous reports revealed that low molecular weight of chitosan and chitosan derivatives exhibited low anti-α-amylase activity, with less than 40% of maximal activity at a dosage of 20 mg/mL^67,68^. Furthermore, the findings indicate that CHQ(1) may have enhanced anti-α-amylase activity through inhibition of α-amylase activity and α-glucosidase as well as increase in glucose uptake. Therefore, the synthesized CHQ derivatives are thought to be promising natural polymers with therapeutic promise in the treatment of diabetes because of their distinct physiochemical properties, wide safety profiles, and variety of advantageous biological activities.

#### In vitro cytotoxicity

As described in Table 4, the in vitro cytotoxicity results of the prepared schiff base derivative on normal HSF cells were shown at different dose concentrations ranging from 25 to 200 mg. A very low level of toxicity was observed for all tested samples in a dose-dependent manner. It was found that about 98.77–99.35% of viable cells were detected using the minimal concentration of samples (*i.e.,* 25 mg). However, a slight increase in toxicity was observed at the highest concentration of 200 mg with maximum values not exceeded than 7.5%, which was recorded by CHQ (1) Schiff base derivative compared to 5.56% for pure chitosan. Preceding studies verified that diverse chitosan Schiff base derivatives had no substantial toxicity towards human cells^60,69^. Since at low doses of the prepared CHQ manifested no antiproliferative impact on the normal HSF cells as CH without any modification, while it can affect the survival of normal HSF cells only if used in elevated doses. Therefore, it is possible to exploit CHQ to produce a therapeutic opportunity for using the prepared CH derivative as therapeutic agent. Thus, up to 200 mg/ml CH and CHQ could be safely used as a antidiabetic agent along with antioxidant and antibacterial efficacy. Overall, these outcomes proved the safety of the synthesized chitosan derivative at the studied concentrations range, suggesting its viability for biomedical applications.

**Table 4:** Cell viability of normal HSF cells treated with chitosan (CH) and its Schiff base derivative (CHQ) with different with different quinoline concentrations.

Conc. (mg)	Viable cells (%)			
	CH	CHQ (0.25)	CHQ (0.5)	CHQ (1)
25	99.35 ± 3.4	99.22 ± 2.3	99.15 ± 3.3	98.77 ± 1.3
50	98.46 ± 2.5	98.34 ± 3.6	98.07 ± 1.8	97.51 ± 3.3
100	97.69 ± 1.7	97.21 ± 3.1	97.05 ± 2.2	96.85 ± 2.1
150	96.84 ± 3.2	96.22 ± 2.8	95.16 ± 1.8	94.45 ± 1.3
200	94.44 ± 2.6	94.13 ± 1.3	94.35 ± 3.7	92.50 ± 2.8

*All data are presented as mean ± SEM of triplicates.

### In silico studies

#### Molecular docking simulations

Molecular docking is a powerful method of examining the interactions between inhibitor molecules and their targets^70^. To determine the binding mode of chitosan (CH) and the newly designed chitosan-quinoline Schiff base derivative (CHQ) at the molecular level, the ligand protein interaction was predicted and estimated using Molecular Operating Environment (MOE) version 2014.09 based on docking score energy represented by binding energy (S). All examined samples were docked inside the active site of α-amylase (PDB: 2QV4) and α-glycosidase (PDB: 3W37). The docking results revealed that the tested CH and CHQ (1) derivative well accommodated in the active site of both α-amylase (Table S1) and α-glycosidase (Table S2) with negative binding energy values.

**Figure 7:**
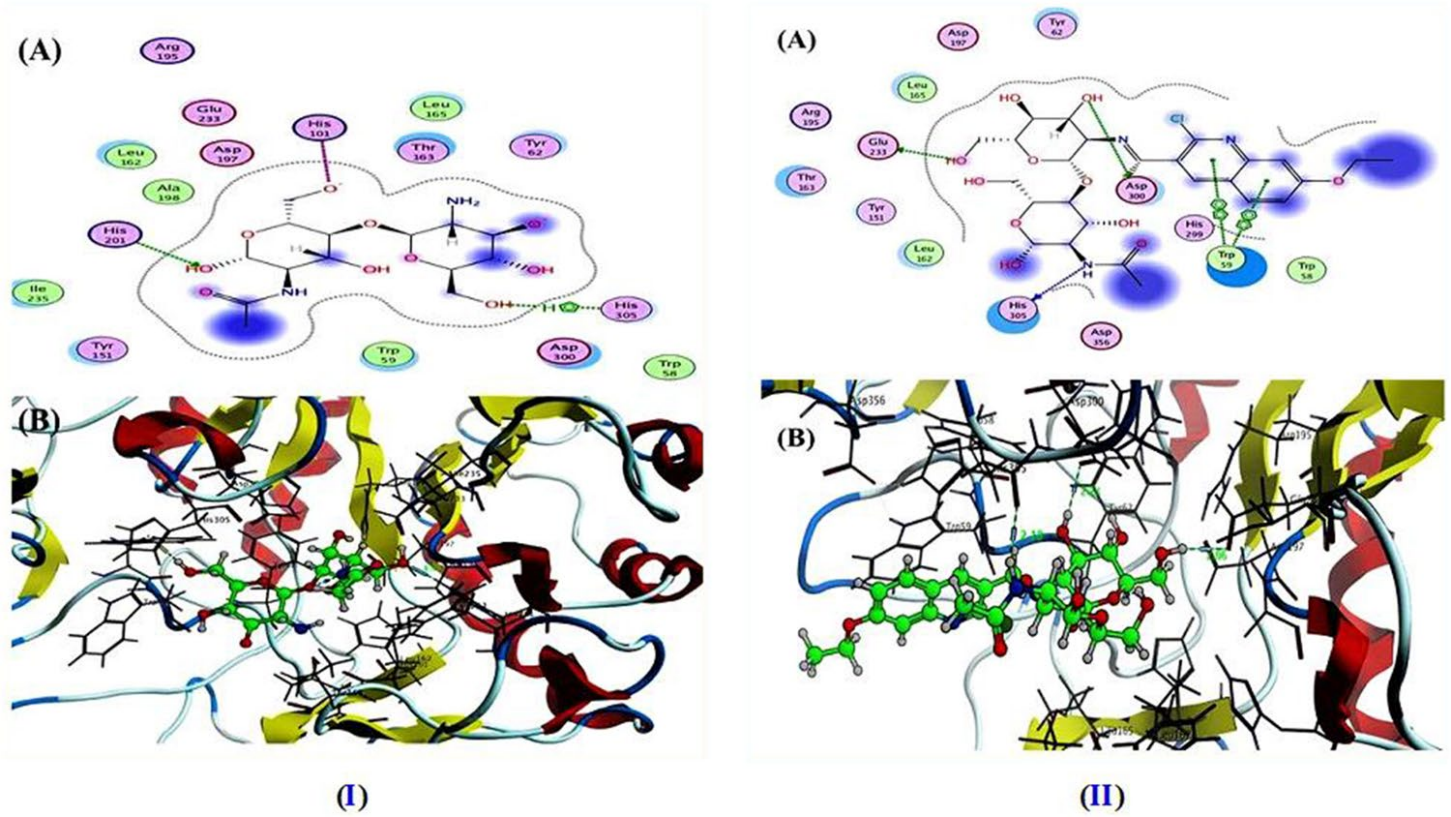
The binding mode and docked pose of (**I**) CH and (**II**) CHQ (1) derivative inside the active site of alpha-amylase (PDB: 2QV4): (**A**) 2D and (**B**) 3D binding interaction.

In the case of α-amylase, the pristine chitosan (CH) (Fig. 7I) displayed binding energy S = − 5.55 kcal/mol with one hydrogen bond side chain acceptor between the residue His201 with oxygen of hydroxy group that attached to C2 of *N*-acetyl glucopyranose ring with a bond length 1.95 Å. In addition to the ionic bond that formed between His101 and oxygen of hydroxy group at C6 of *N*-acetyl glucopyranose ring. Further, the hydroxy group at C6 glucopyranose ring formed arene-H interaction with the residue His305. Furthermore, the chitosan-quinoline derivative (CHQ) depicted in Fig. 7II exposed binding energy (S) equal to − 6.79 kcal/mol through three hydrogen bonds and one arene-arene interaction. Two of three hydrogen bonds belong to sidechain hydrogen bond donor between the residue Glu233 and Asp300 and hydrogen of hydroxy group at C6 and C3 of glucopyranose ring with bond length 2.06 and 2.33 Å. Besides, one hydrogen bond backbone donor between the residue His305 and NH of acetanilide in *N*-acetyl glucopyranose ring of chitosan structure with a bond length reached 2.18 Å, as well as arene-arene interaction between Trp59 and quinoline ring (phenyl and pyridine). Besides, the acarbose (ACA; as co-crystallized ligand) exhibited binding energy S = − 9.62 kcal/mol with RMSD = 1.095 Å through two hydrogen bonds sidechain acceptors (His299 and His201) and five hydrogen bonds sidechain donor (Asn105, Asp300, and Glu233). All docking figures in full size were represented in the supplementary material file.

**Figure 8:**
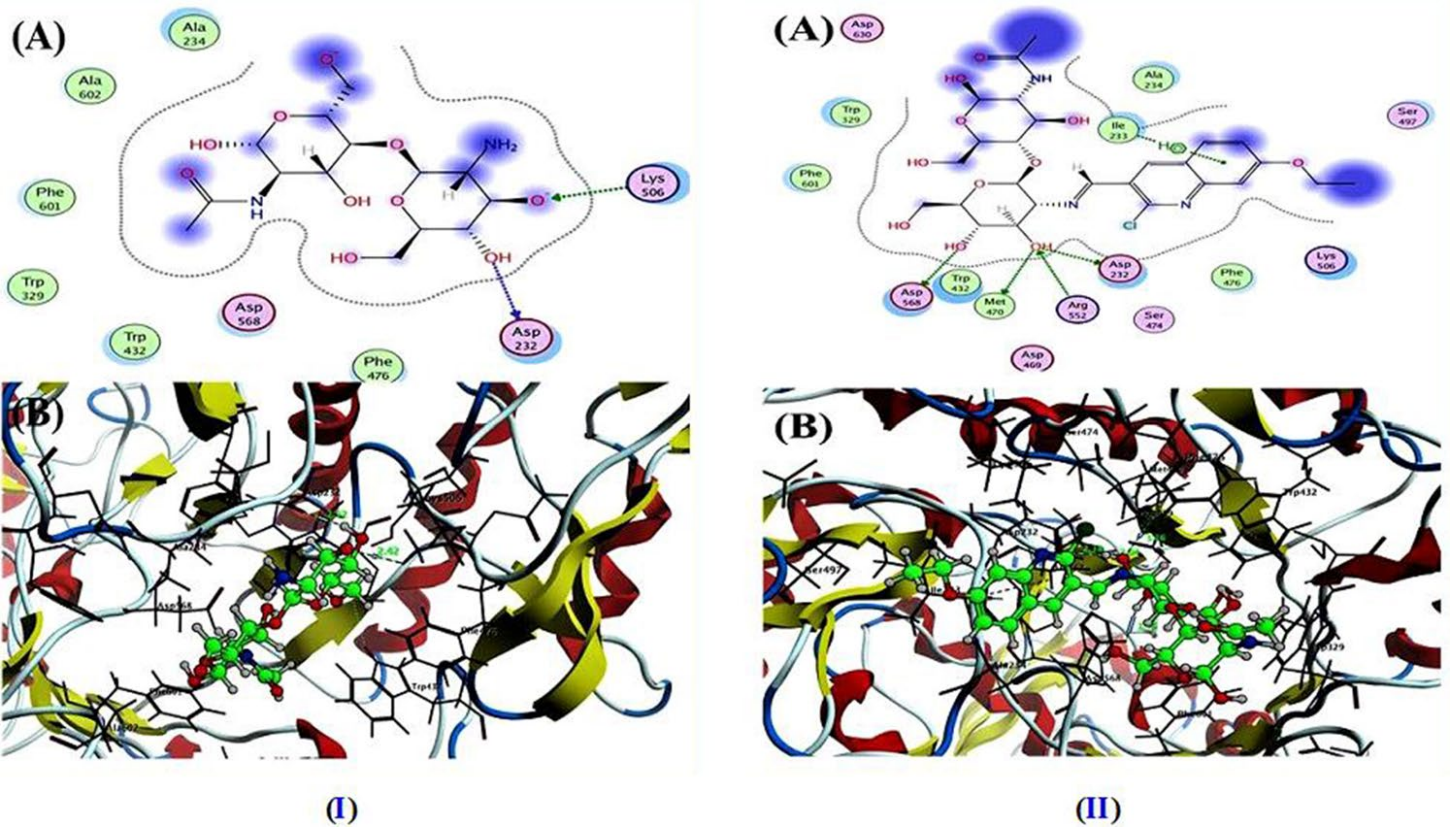
The binding mode and docked pose of (**I**) CH and (**II**) CHQ (1) derivative inside the active site of alpha-glucosidase (PDB: 3W37): (**A**) 2D and (**B**) 3D binding interaction.

On the other hand, the ability of CH and CHQ derivative derivatives to inhibit alpha-glycosidase was investigated to explore their potential as antidiabetic agents. The molecular docking simulation of the tested samples was determined with lower binding affinity, and these results are represented in Table S2 and Fig. 8I and II. The docking results showed well correlated with the experimental data inside the active site of alpha-glycosidase (PDB: 3W37). With the docking score ranging from − 6.63 to − 4.78 kcal/mol, the synthesized CH and CHQ derivative had excellent interaction with the target enzyme. Unexpectedly, the CHQ Schiff base derivative demonstrated binding energy S = − 6.63 kcal/mol higher than pure chitosan (CH) (S = − 4.78 kcal/mol). The CHQ also displayed four hydrogen bonds interactions in the target active site classified three hydrogen bonds sidechain donor (Asp232, Met470, and Asp568) and one hydrogen bond sidechain acceptor (Agr552) with bond length 2.18, 3.16, 1.95, and 2.04 ˚A, respectively. Besides, one arene-H interaction is present between the residue Ile233 and phenyl of quinoline derivative. It is possible that these interactions are essential for maintaining the stability of the ligand-target complex. Conversely, when the chitosan (CH) was docked in the enzymes active site, it revealed binding energy S = − 4.78 kcal/mol by establishing two hydrogen bonds with Asp232 and Lys506 at distance of 2.12 and 2.42 Å. Further, the Co-crystallized ligand (ACA) containing a number of hydroxy and methyl hydroxy groups that have ability to demonstrate a greater number of hydrogen bonds interaction inside the active site. In addition, the ACA displayed binding energy S = − 8.69 kcal/mol with RMSD = 2.05 Å through eight hydrogen bonds sidechain donor and two hydrogen bonds side-chain acceptor.

#### DFT computational studies

**Figure 9:**
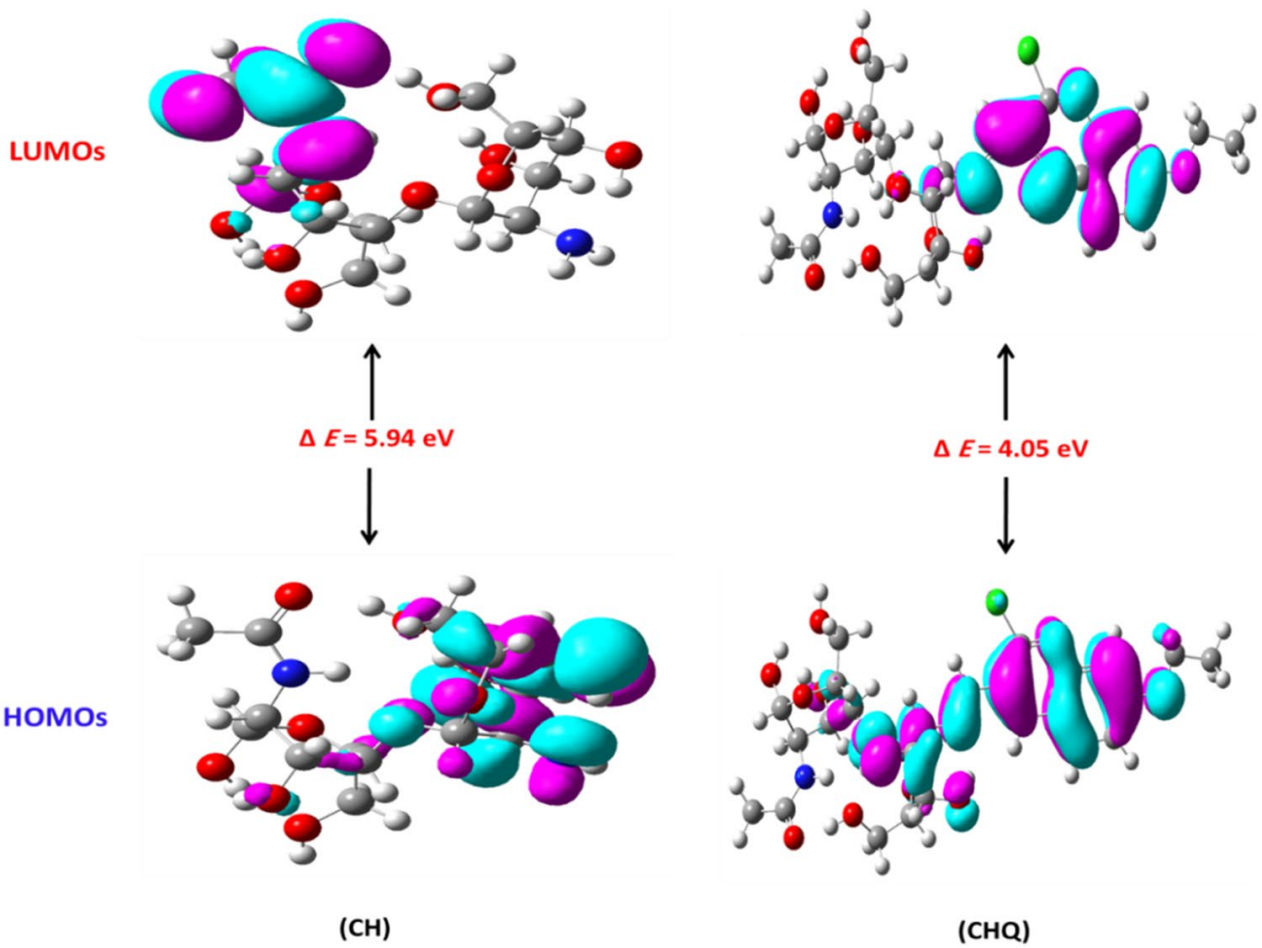
Electron density of the molecular FMOs with energy band gap values for CHQ (1) derivative.

The frontier molecular orbitals (FMO) of compounds demonstrate the critical role of charge-transfer interactions with the active site of binding proteins^71^. The suggested model to build model molecules consisted of two subunits for chitosan, with one quinoline unit as blend were studied. Our work involved study of the molecular orbitals for CH and CHQ (1) derivative and the molecular model molecules were represented in Fig. S3. The frontier molecular orbitals feature the highest occupied (HOMO) and lowest unoccupied (LUMO) orbitals. A transition from the ground state to the first excited state is termed electronic absorption, which is mainly represented by one electron excited from HOMO to LUMO. The difference between the HOMO and LUMO is known as the energy band gap (Δ E) and molecules with extended energy gaps have high chemical stability and low chemical reactivity^72^. Furthermore, The HOMO energy of a molecule indicates its ability to donate electrons, while the LUMO energy indicates its ability to accept electrons^73^. The calculated FMO energies and chemical reactivity descriptors values of optimized geometry were listed in Table 5 and Fig. 9. The positive lopes are presented in turquase color, while the negative lopes is expresed in pale purple color.

**Table 5:** The FMO energies and chemical reactivity descriptors values of CH and CHQ (1) derivative.

FMO and reactivity descriptors		CH	CHQ
Parameters	Symbol (Unit)		
Total energy	E_Total_ (Hartree)	− 1410.81	− 2463.09
Diploe moment	µ (Debye)	6.41	5.71
HOMO energy	E_HOMO_ (eV)	− 6.39	− 6.19
LUMO energy	E_LUMO_ (eV)	− 0.44	− 2.15
Energy gap	ΔE (eV)	5.94	4.05
Ionization potential	ӀP (eV)	6.39	6.19
Electron affinity	EA (eV)	0.44	2.15
Electronegativity	X (eV)	3.42	4.18
Chemical hardness	ɳ (eV)	2.97	2.02
Chemical softness	S (eV^−1^)	0.34	0.49
Chemical potential	µp (eV)	− 3.42	− 4.18
Electrophilic index	ω	1.96	4.31

The chitosan (CH) HOMO orbital which indicate nucleophilic attach site is mainly distributed over the glucopyranose ring (GlcN) only and ether linkage, while the LUMO orbital spread over the acetyl group of *N*-acetyl glucopyranose ring (GlcNAc).In addition, the HOMO electron density of chitosan-quinoline derivative (CHQ) is mainly localized on quinoline moiety, imino group (C=N), and glucopyranose (GlcN) except C6 (-CH_2_OH), while the LUMO orbital located on quinoline moiety and imino group (C=N). Surprisingly, the CHQ (1) derivative showed the lowest energy band gap (ΔE) = 4.05 eV compared to native chitosan (ΔE = 5.94 eV).

The tested compounds displayed low total energy ranging from − 1128.69 to − 2463.09 Hartree. Introducing the quinoline scaffold to chitosan to afford Schiff base derivatives (CHQ) increases the stability of the newly designed derivative with increased the total energy E total = − 2463.09 Hartree. Additionally, these derivatives displayed higher dipole moment values ranging between (5.04 and 6.41) Debye, where molecules with high dipole moment values are more likely to be involved in intermolecular interactions. Moreover, the high dipole moment value indicates a more polar nature^74^. The reactivity descriptors obtained from Koopman's theorem could be used to identify a molecule's reactivity as ionization potential (i); electron affinity (EA); electronegativity (X); chemical hardness (ɳ); chemical softness (S); chemical potential (µp); electrophilic index (**ω**) that calculated according to the previously reported method^53^.

**Figure 10:**
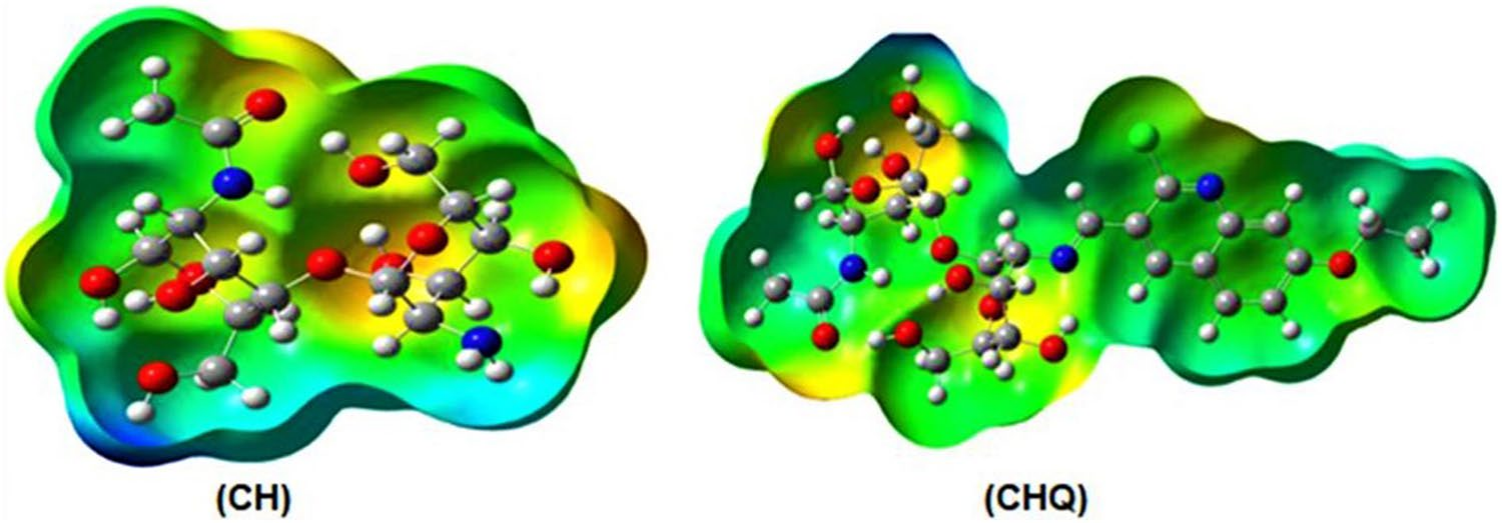
Molecular electrostatic potential surface of CH and CHQ (1) derivative using the DFT/B3LYP calculation.

Besides, Fig. 10 describes the molecular electrostatic potential (MEP) surface of CH and CHQ derivatives using the DFT/B3LYP calculation. Factually, the molecular electrostatic potential (MEP) is one of the most effective ways of analyzing the chemical reactivity of a molecule against positively or negatively charged reagents. MEP analysis is a method for determining the position in the molecule that has the ability to form hydrogen bonds, intramolecular, and intermolecular interactions inside the active site in the pocket and confirmed the docking simulation study^51^. The electrostatic potential value of a molecule is determined by the difference in color distributed over the 3D structure and increases in the order blue > green > yellow > orange > red. The red color in MEP pointed to a more electron-rich region, while the blue color indicated an electron-poor region. The neutral electrostatic potential is represented by a green and yellow color, which is localized mainly on phenyl of quinoline, ethyl of ethoxy, and methylene group of hydroxy methyl derivative (-CH_2_OH) at the glucopyranose ring. The negative potential regions are localized over nitrogen and oxygen atoms, while the positive potential region was found over hydrogen atoms of the amino (NH_2_) group and hydroxy methyl at C6 of glucopyranose derivative.

Finally, the results concluded that forming a Schiff base based on chitosan-quinoline derivatives significantly enhanced the biological activities of the pristine chitosan. Also, it is thought that these activities can be attributed to the difference in electrostatic potential distribution around the drug and the difference in the energies of HOMO, LUMO, and energy bandgap. As well, the MEPs showed the positions in the molecules that are responsible for activity by forming different types of interactions inside the active site of the pocket.

## Conclusion

In summary, a new multi-featured chitosan-quinoline Schiff base derivative was synthetized as potent. Maximal degree of substitution was 78.83% and recorded by the highest quinolone concentration (*i.e.,* 1 M). The results denoted also that increasing the quinolone ratio had a positive impact on antibacterial activity with maximum inhibition of 97.55 and 75.44% against *S. haemolyticus* and *E. coli* compared to 57.3 and 36.9% for pristine chitosan. The antioxidant activity of chitosan was also boosted after the Schiff base formation. Furthermore, the CHQ (1) derivative proved its competence in inhibiting both α-amylase and α-glucosidase with maximal inhibitory values reached 99.78 and 92.10%, respectively. Besides, the molecular docking studies were performed to rationalize the perceived antidiabetic activity and proved the high stability of CHQ (1) in the active sites of α-amylase and α-glucosidase. The in vitro cytotoxicity assessment revealed the biosafety of the synthesized CHQ (1) derivative. In conclusion, the gained multi-bio-characteristics of the designed chitosan-quinoline Schiff base derivative implying its future practical use for biomedical applications, predominantly as safe and powerful antibacterial as well as antioxidant agent for accelerating wound healing. It can be also applied as diabetes mellitus drug for regulation the blood glucose level in addition to inhibiting both α-amylase and α-glucosidase in diabetic patients.

### Supplementary Information

Supplementary Information.

## Data Availability

The datasets used and/or analysed during the current study are available from the corresponding author on reasonable request.
