# Supplementary material for: Developing a new multi-featured chitosan-quinoline Schiff base with potent antibacterial, antioxidant, and antidiabetic activities: design and molecular modeling simulation
Source: Sci Rep. 2023 Dec 21;13:22792. doi: 10.1038/s41598-023-50130-3 (PMC10733428; doi:10.1038/s41598-023-50130-3)
Supplement: Supplementary file 1 — Supplementary Information. [file 41598_2023_50130_MOESM1_ESM.docx]

**Supplementary information**

**Developing a new multi-featured chitosan-quinoline Schiff base with potent antibacterial, antioxidant, and antidiabetic activities: Design and molecular modeling simulation**

Yasser M. Abdel-Baky^1^, Ahmed M. Omer ^2,^ *, Esmail M. El-Fakharany ^3^, Yousry A. Ammar^1^, Moustafa S. Abusaif ^1^, Ahmed Ragab ^1,*^

^1^ Chemistry Department, Faculty of Science, Al-Azhar University, Nasr City 11884 Cairo, Egypt

^2^ Polymeric Materials Research Department, Advanced Technology and New Materials Research Institute (ATNMRI), City of Scientific Research and Technological Applications (SRTA-City), New Borg El-Arab City, P. O. Box: 21934, Alexandria, Egypt

^3^ Protein Research Department, Genetic Engineering and Biotechnology Research Institute, City of Scientific Research and Technological Applications (SRTA-City), New Borg El-Arab City, P. O. Box: 21934, Alexandria, Egypt

* Correspondence: Ahmed M. Omer ([amomar@srtacity.sci.eg](mailto:amomar@srtacity.sci.eg)), Ahmed Ragab ([ahmed_ragab7@ymail.com](mailto:ahmed_ragab7@ymail.com);ahmed_ragab@azhar.edu.eg)

**Table (S1):** Binding energy (S) represented by (Kcal/mol) and docking analysis of the most active derivatives inside the active site of alpha-amylase (PDB: 2QV4).

| Cpd  No. | S (Kcal/mol) | Amino acid residues | Distance  °A | Type of  interaction | Hydrophobic interaction |
| --- | --- | --- | --- | --- | --- |
| CH | -5.55 | His201  His305  His101 | 1.95  -  i | SCA  -  i | Leu165, Thr163, Tyr62, Arg195, Glu233, Asp197, Leu162, Ala198, Ile235, Tyr151, Trp59, Asp300, Trp58 |
| CHQ | -6.79 | Glu233  Asp300  His305  Trp59 | 2.06  2.23  2.18  -- | SCD  SCD  BBA  -- | Tyr62, Asp197, Leu165, Arg195, Thr163, Tyr151, Leu162, Asp356, Try58, His299 |
| ACA | -9.62 | His299  His201  Asn105  Asp300  Asp300  Asp300  Glu233 | 2.04  1.83  2.12  1.78  2.15  2.57  2.06 | SCA  SCA  SCD  SCD  SCD  SCD  SCD | Asp197, Ile235, Leu165, His101, Val107, Leu162, Gly164, Ala106, Thr163, Trp59, Tyr62, Trp58, His305, Gln63, Arg195, Ala198, Tyr151 |

**ACA**: Acarbose as co-crystalized ligand with RMSD=1.098 °A; **CH**: chitosan; **CHQ**: chitosan-quinoline derivative; **SCD**: sidechain donor; **SCA**: sidechain acceptor; **BBD**: backbone donor; **BBA**: backbone acceptors; (**-**): arene-hydrogen interaction; (i): ionic bond interaction; (--): arene-arene interaction.

**Table S2**. Binding energy (S) represented by (Kcal/mol) and docking analysis of the most active derivatives inside the active site of alpha-glycosidase (PDB: 3W37).

| Cpd  No. | S (Kcal/mol) | Amino acid residues | Distance  °A | Type of  interaction | Hydrophobic interaction |
| --- | --- | --- | --- | --- | --- |
| CH | -4.78 | Asp232  Lys506 | 2.12  2.42 | BBD  SCA | Ala234, Ala602, Phe601, Trp329, Trp432, Asp568, Phe476 |
| CHQ | -6.63 | Asp232  Arg552  Met470  Asp568  Ile233 | 2.18  2.04  3.16  1.95  - | SCD  SCA  SCD  SCD  - | Asp630, Trp329, Phe601, Trp423, Asp469, Ser474, Phe476, Lys506, Ser497, Ala234 |
| ACA | -8.69 | Asp232  Asp232  Met470  Arg552  Asp568  Asp568  Asp568  His626  Asp357  Asp357 | 2.10  2.06  3.17  1.90  2.47  1.76  1.79  2.33  1.97  1.95 | SCD  SCD  SCA  SCD  SCD  SCD  SCA  SCD  SCD  SCD | Ile396, Ile358, Trp467, Trp329, Asn237, Phe604, Trp565, Asn469, Trp423, Ile233, Phe476, Ala234, Phe236, Ser497 |

**ACA** : Acarbose as co-crystalized ligand with RMSD= 2.69 °A; **CH**; chitosan; **CHQ**: chitosan-quinoline derivative; **SCD**: sidechain donor; **SCA**: sidechain acceptor; **BBD**: backbone donor; **BBA**: backbone acceptors; (-): arene-hydrogen interaction.


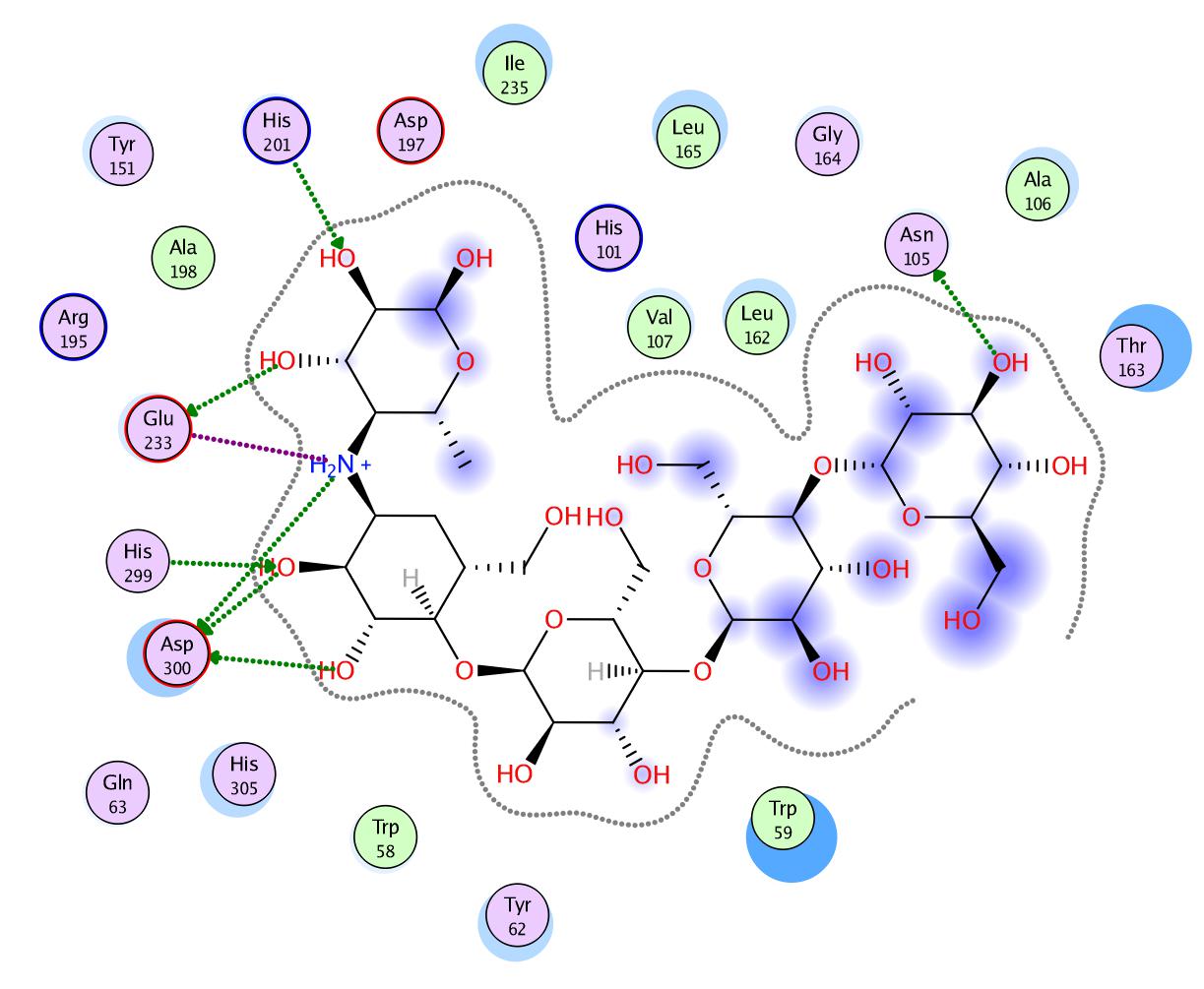


**Fig. S1 (A):** 2D binding mode and docked pose of **co-crystallized ligand** derivative inside the active site of alpha-amylase (PDB: 2QV4).


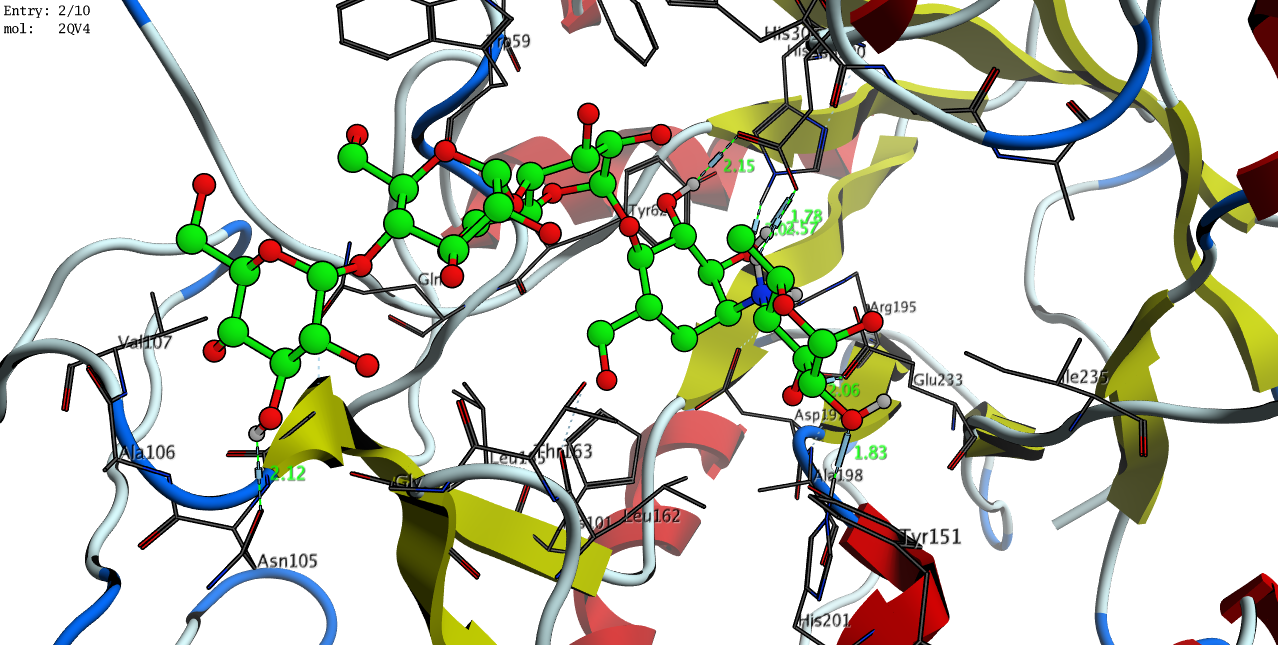


**Fig. S1 (B):** 3D binding mode and docked pose of **co-crystallized ligand** derivative inside the active site of alpha-amylase (PDB: 2QV4).


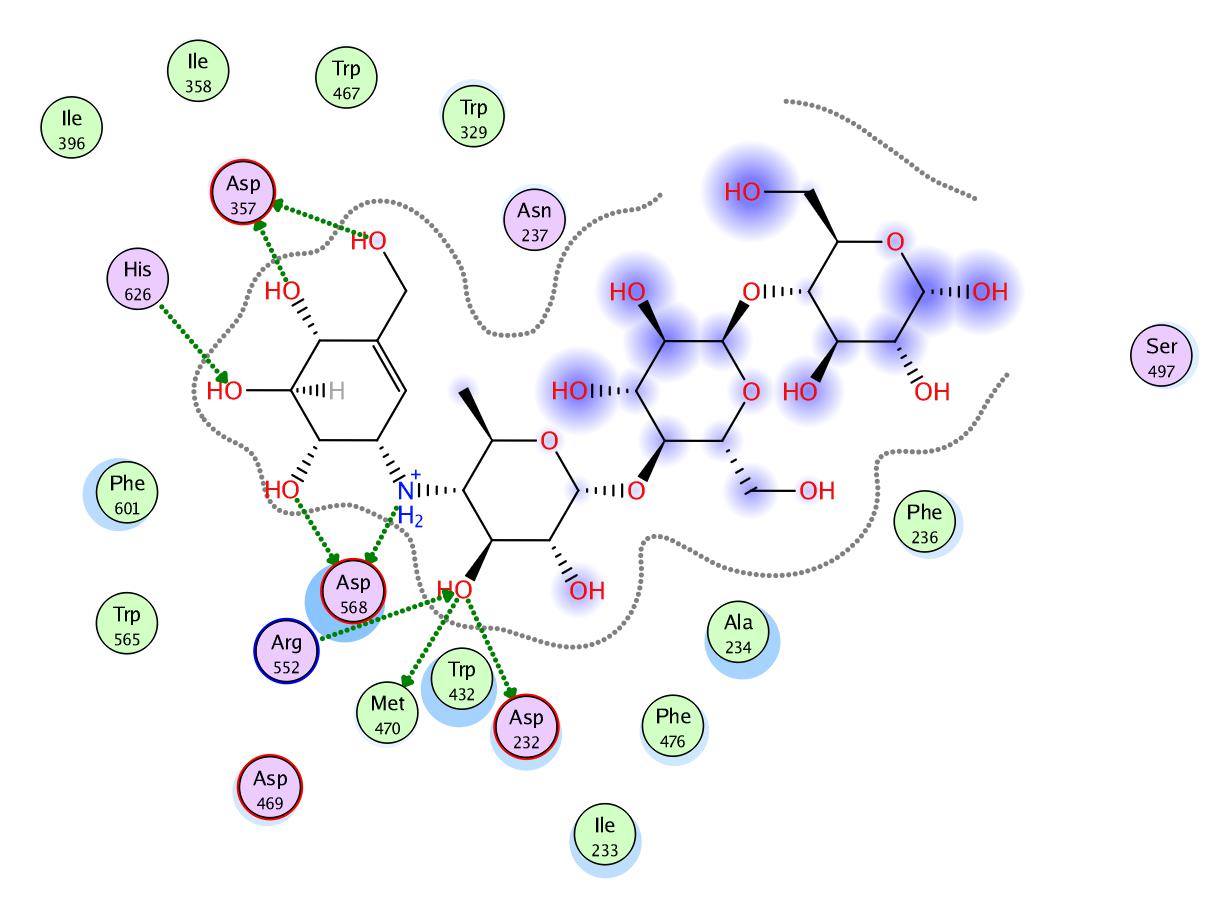


**Fig. S2 (A):** 2D binding mode and docked pose of **co-crystallized ligand** derivative inside the active site of alpha-glucosidase (PDB: 3W37).


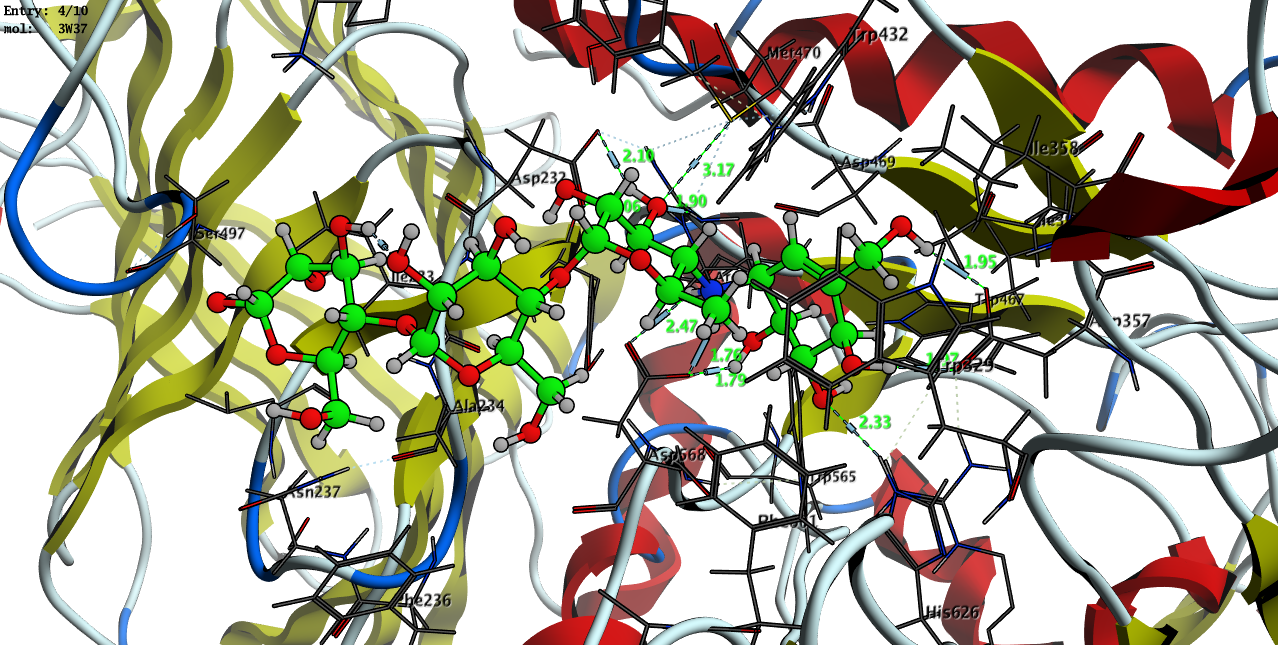


**Fig. S2 (B):** 3D binding mode and docked pose of **co-crystallized ligand** derivative inside the active site of alpha-glucosidase (PDB: 3W37).


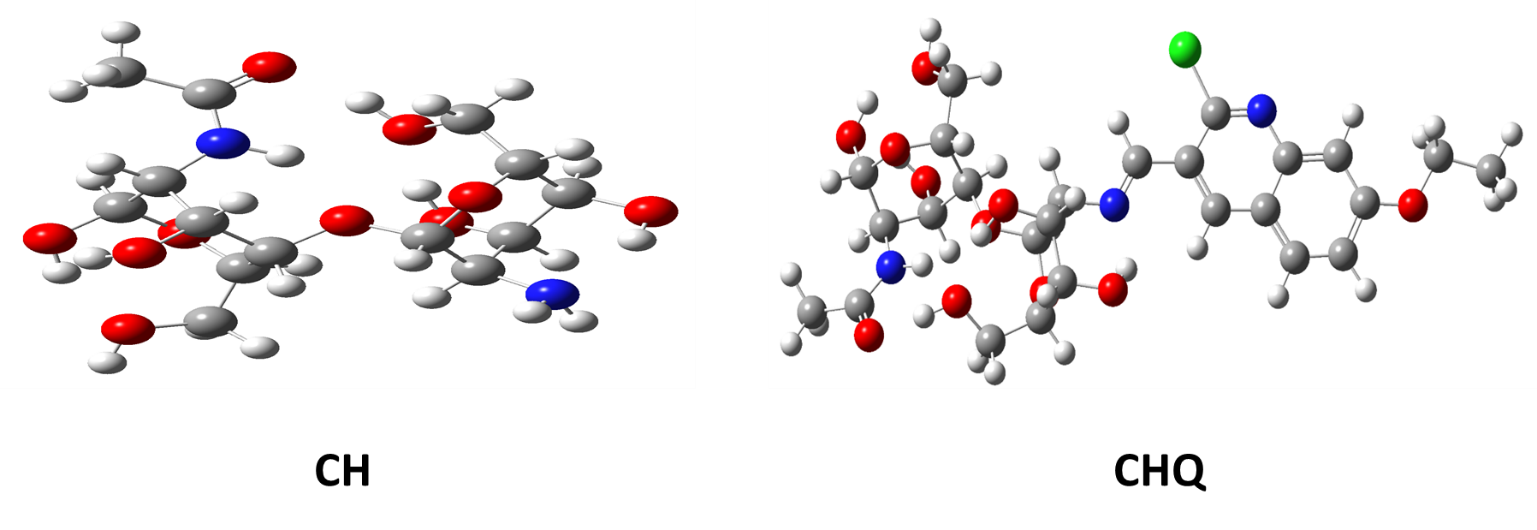


**Fig. S3.** Molecular modeling molecules of **CH** and **CHQ** derivative.
